# Supplementary material for: Prevalence of symptom exaggeration among North American independent medical evaluation examinees: A systematic review of observational studies
Source: PLoS One. 2025 Jun 25;20(6):e0324684. doi: 10.1371/journal.pone.0324684 (PMC12193048; doi:10.1371/journal.pone.0324684)
Supplement: S1 Table — (DOCX) [file pone.0324684.s001.docx]

### S1 Table: Search Strategies

**MEDLINE**

Database: OVID Medline Epub Ahead of Print, In-Process & Other Non-Indexed Citations, Ovid MEDLINE(R) Daily and Ovid MEDLINE(R) 1946 to Present

Search Strategy:

--------------------------------------------------------------------------------

1 (independent adj3 medical adj3 exam$).ti,ab. (93)

2 (independent adj3 medical adj3 evaluat$).ti,ab. (69)

3 1 or 2 (159)

4 Physical Examination/ (42139)

5 Disability Evaluation/ (50139)

6 Work Capacity Evaluation/ (6169)

7 or/4-6 (96526)

8 independent.ti,ab. (942148)

9 7 and 8 (3901)

10 3 or 9 (4002)

11 ("classification accuracy" or disability or disabled or detection or detecting or detect or validity or validation or effort or "known groups" or performance or MMPI or "independent medical evaluation" or "independent medical examination").mp. [mp=title, abstract, original title, name of substance word, subject heading word, floating sub-heading word, keyword heading word, organism supplementary concept word, protocol supplementary concept word, rare disease supplementary concept word, unique identifier, synonyms] (3136710)

12 (litigant or litigation or litigant or incentive or claimant* or "fake bad" or exaggerat* or "brain injury" or "brain damage" or "head injury" or "personal injury" or neuropsychologic* or neurocognit*).mp. [mp=title, abstract, original title, name of substance word, subject heading word, floating sub-heading word, keyword heading word, organism supplementary concept word, protocol supplementary concept word, rare disease supplementary concept word, unique identifier, synonyms] (304834)

13 (malinger* or litigation or litigant or "insufficient effort").mp. (10557)

14 11 and 12 and 13 (2015)

15 10 or 14 (5982)

**EMBASE (OVID)**

Database: Embase <1974 to 2021 October 25>

Search Strategy:

--------------------------------------------------------------------------------

1 (independent adj3 medical adj3 exam$).ti,ab. (107)

2 (independent adj3 medical adj3 evaluat$).ti,ab. (80)

3 1 or 2 (185)

4 physical examination/ (237066)

5 disability/ (115825)

6 work capacity/ (12956)

7 or/4-6 (363277)

8 independent.ti,ab. (1247976)

9 7 and 8 (11281)

10 3 or 9 (11419)

11 ("classification accuracy" or disability or disabled or detection or detecting or detect or validity or validation or effort or "known groups" or performance or MMPI or "independent medical evaluation" or "independent medical examination").mp. [mp=title, abstract, heading word, drug trade name, original title, device manufacturer, drug manufacturer, device trade name, keyword heading word, floating subheading word, candidate term word] (4344778)

12 (litigant or litigation or litigant or incentive or claimant* or "fake bad" or exaggerat* or "brain injury" or "brain damage" or "head injury" or "personal injury" or neuropsychologic* or neurocognit*).mp. [mp=title, abstract, heading word, drug trade name, original title, device manufacturer, drug manufacturer, device trade name, keyword heading word, floating subheading word, candidate term word] (445562)

13 (malinger* or litigation or litigant or "insufficient effort").mp. (12718)

14 11 and 12 and 13 (2425)

15 10 or 14 (13805)

**PsycInfo (OVID)**

Database: APA PsycInfo <1806 to October Week 3 2021>

Search Strategy:

--------------------------------------------------------------------------------

1 (independent adj3 medical adj3 exam$).ti,ab. (51)

2 (independent adj3 medical adj3 evaluat$).ti,ab. (31)

3 1 or 2 (80)

4 physical examination/ (1145)

5 Disability Evaluation/ (619)

6 work capacity evaluation$.ti,ab. (6)

7 or/4-6 (1765)

8 independent.ti,ab. (160675)

9 7 and 8 (88)

10 3 or 9 (149)

11 ("classification accuracy" or disability or disabled or detection or detecting or detect or validity or validation or effort or "known groups" or performance or MMPI or "independent medical evaluation" or "independent medical examination").mp. [mp=title, abstract, heading word, table of contents, key concepts, original title, tests & measures, mesh] (840550)

12 (litigant or litigation or litigant or incentive or claimant* or "fake bad" or exaggerat* or "brain injury" or "brain damage" or "head injury" or "personal injury" or neuropsychologic* or neurocognit*).mp. [mp=title, abstract, heading word, table of contents, key concepts, original title, tests & measures, mesh] (180968)

13 (malinger* or litigation or litigant or "insufficient effort").mp. (8875)

14 11 and 12 and 13 (2842)

15 10 or 14 (2964)

**CINAHL**

| **#** | **Query** | **Results** |
| --- | --- | --- |
| S17 | S15 AND S16 | 117 |
| S16 | EM 202010- | 385,047 |
| S15 | S10 OR S14 | 2,742 |
| S14 | S11 AND S12 AND S13 | 839 |
| S13 | TX malinger* or litigation or litigant or "insufficient effort" | 4,668 |
| S12 | TX litigant or litigation or litigant or incentive or claimant* or "fake bad" or exaggerat* or "brain injury" or "brain damage" or "head injury" or "personal injury" or neuropsychologic* or neurocognit* | 126,827 |
| S11 | TX "classification accuracy" or disability or disabled or detection or detecting or detect or validity or validation or effort or "known groups" or performance or MMPI or "independent medical evaluation" or "independent medical examination" | 1,057,306 |
| S10 | S6 OR S9 | 1,912 |
| S9 | S7 AND S8 | 1,859 |
| S8 | TX independent | 191,131 |
| S7 | S3 OR S4 OR S5 | 46,812 |
| S6 | S1 OR S2 | 92 |
| S5 | (MH "Work Capacity Evaluation") | 1,691 |
| S4 | (MH "Disability Evaluation") | 16,301 |
| S3 | (MH "Physical Examination") | 29,240 |
| S2 | TX independent N3 medical N3 evaluat* | 40 |
| S1 | TX independent N3 medical N3 exam* | 55 |
