# Supplementary material for: Prevalence of symptom exaggeration among North American independent medical evaluation examinees: A systematic review of observational studies
Source: PLoS One. 2025 Jun 25;20(6):e0324684. doi: 10.1371/journal.pone.0324684 (PMC12193048; doi:10.1371/journal.pone.0324684)
Supplement: S2 Table — (DOCX) [file pone.0324684.s002.docx]

### S2 Table: Risk of bias assessment

| Author | Year | Does the sample either represent a consecutive or random sample of a group of IME referrals?  Yes (not serious risk of bias)  No (serious risk of bias) | Was the index test administered without knowledge of the reference standard? | Missing data  (>20% = Serious risk of bias) | Was age and education among target positive and target negative groups similar? | Confidence in reference standard |
| --- | --- | --- | --- | --- | --- | --- |
| Aguerrevere, | 2008 | Yes (Consecutive sample) | No | <5% | Yes | Moderate |
| Aguerrevere | 2011 | Yes (Consecutive sample) | No | <5% | Yes | Moderate |
| Aguerrevere | 2017 | Yes (Consecutive sample) | No | <5% | Yes | Moderate |
| Ardolf | 2007 | Yes (Consecutive sample) | No | <5% | Yes | Moderate |
| Barrash | 2004 | Yes (Consecutive sample) | No | <5% | Yes | Strong |
| Bianchini | 2014 | Yes (Consecutive sample) | No | <5% | Not reported | Moderate |
| Bianchini | 2018 | Yes (Consecutive sample) | No | <5% | Yes | Moderate |
| Bortnik | 2010 | Yes (Consecutive sample) | No | <5% | Yes | Moderate |
| Costa | 1999 | Yes (Consecutive sample) | No | <5% | Yes | Weak |
| Curtis | 2006 | Yes (Consecutive sample) | No | <5% | Not reported | Moderate |
| Curtis | 2008 | Yes (Consecutive sample) | No | <5% | Yes | Moderate |
| Curtis | 2010 | Yes (Consecutive sample) | No | <5% | Yes | Moderate |
| Curtis | 2019 | Yes (Consecutive sample) | No | <5% | Yes | Moderate |
| Etherton | 2006a | Yes (Consecutive sample) | No | <5% | Yes | Weak |
| Greve | 2003 | Yes (Consecutive sample) | No | <5% | Yes | Moderate |
| Greve | 2006a | Yes (Consecutive sample) | No | <5% | Not reported | Moderate |
| Greve | 2006b | Yes (Consecutive sample) | No | <5% | Not reported | Moderate |
| Greve | 2006c | Yes (Consecutive sample) | No | <5% | Not reported | Moderate |
| Greve | 2006d | Yes (Consecutive sample) | No | <5% | Not reported | Moderate |
| Greve | 2007 | Yes (Consecutive sample) | No | <5% | Yes | Moderate |
| Greve | 2007a | Yes (Consecutive sample) | No | <5% | Yes | Moderate |
| Greve | 2008b (TBI and chronic pain cohorts) | Yes (Consecutive sample) | No | <5% | No | Moderate |
| Greve | 2008 | Yes (Consecutive sample) | No | <5% | Yes | Moderate |
| Greve | 2009 | Yes (Consecutive sample) | No | <5% | Not reported | Moderate |
| Greve | 2009 | Yes (Consecutive sample) | No | <5% | Yes | Moderate |
| Greve | 2009a | Yes (Consecutive sample) | No | <5% | Not reported | Moderate |
| Greve | 2009c | Yes (Consecutive sample) | No | <5% | No | Moderate |
| Greve | 2009d | Yes (Consecutive sample) | No | <5% | Not reported | Moderate |
| Greve | 2009 (TBI and chronic pain cohorts) | Yes (Consecutive sample) | No | <5% | Not reported | Weak |
| Greiffenstein | 1995 | Yes (Consecutive sample) | No | <5% | No | Strong |
| Greve | 2010 | Yes (Consecutive sample) | No | <5% | No | Weak |
| Guise | 2014 | Yes (Consecutive sample) | No | <5% | Yes | Moderate |
| Heinly | 2005 | Yes (Consecutive sample) | No | <5% | Yes | Moderate |
| Henry | 2007 | Yes (Consecutive sample) | No | <5% | Yes | Moderate |
| Henry | 2009 | Yes (Consecutive sample) | No | <5% | Yes | Moderate |
| Lees-Haley | 1991 | Yes (Consecutive sample) | No | <5% | Not reported | Moderate |
| Lu | 2003 | Yes (Consecutive sample) | No | <5% | Yes | Strong |
| O'Bryant | 2007 | Yes (Consecutive sample) | No | <5% | Not reported | Moderate |
| Ord | 2008 | Yes (Consecutive sample) | No | <5% | Yes | Moderate |
| Ord | 2010 | Yes (Consecutive sample) | No | <5% | Yes | Moderate |
| Patrick | 2014 | Yes (Consecutive sample) | No | <5% | Yes | Moderate |
| Roberson | 2013 | Yes (Consecutive sample) | No | <5% | Yes | Moderate |
| Suhr | 1997 | Yes (Consecutive sample) | No | <5% | Yes | Strong |
| Sweet | 2000 | Yes (Consecutive sample) | No | <5% | Yes | Strong |
| van Gorp | 1999 | Yes (Consecutive sample) | No | <5% | Yes | Moderate |
