# Supplementary material for: Prevalence of symptom exaggeration among North American independent medical evaluation examinees: A systematic review of observational studies
Source: PLoS One. 2025 Jun 25;20(6):e0324684. doi: 10.1371/journal.pone.0324684 (PMC12193048; doi:10.1371/journal.pone.0324684)
Supplement: S3 Table — (DOCX) [file pone.0324684.s003.docx]

**S3 Table:** ICEMAN criteria to assess credibility of subgroup effect of female % and prevalence

| ICEMEN Criteria | Female% |
| --- | --- |
| 1: Is the analysis of effect modification based on comparison within rather than between trials? | Between-study |
| 2: For within-trial comparisons, is the effect modification similar from trial to trial? | NA |
| 3: For between-trial comparisons, is the number of trials large? | Rather large (9 studies in the smallest subgroup) |
| 4: Was the direction of effect modification correctly hypothesized a priori? | Yes |
| 5: Does a test for interaction suggest that chance is an unlikely explanation of the apparent effect modification? | Unclear (p=0.02) |
| 6: Did the authors test only a small number of effect modifiers or consider the number in their statistical analysis? | Unclear |
| 7: Did the authors use a random effects model? | Definitely yes |
| 8: If the effect modifier is a continuous variable, were arbitrary cut points avoided? | No |
| 9 Optional: Are there any additional considerations that may increase or decrease credibility? | NA |
| The effect modification persisted after adjustment for other potential effect modifiers | NA |
| The effect modification is consistent across related outcomes: | NA |
| A sensitivity analysis suggested robustness to relevant assumptions | NA |
| Effect modification supported by external evidence | Yes |
| “Dose-response effect” across levels of the effect modifier | Yes |
| Risk of bias of the main effects of the individual RCTs or the meta-analysis | NA |
| The meta-analysis had had exceptionally high power to detect the effect modification | No |
| Overall credibility | Low-Moderate* |

ICEMAN: Instrument for assessing the Credibility of Effect Modification Analyses; NA: not applicable

*For the number of trials, we just fell below the threshold of 10 per subgroup and for test of interaction we just fell below threshold of p=0.01. Accordingly, we selected a credibility of low to moderate.
