# Supplementary material for: Prevalence of symptom exaggeration among North American independent medical evaluation examinees: A systematic review of observational studies
Source: PLoS One. 2025 Jun 25;20(6):e0324684. doi: 10.1371/journal.pone.0324684 (PMC12193048; doi:10.1371/journal.pone.0324684)
Supplement: S4 Table — (DOCX) [file pone.0324684.s004.docx]

**S4 Table:** Psychometric properties of tests included in symptom exaggeration criteria with list of references

| **Criteria** | **Scale** | **Psychometric Properties** |
| --- | --- | --- |
| **Criteria from Slick et al., Performance Validity Tests (PVTs)** | | |
| Test of Memory Malingering (TOMM) | NA | **Cohen, 2022 (46)**   - **Study Characteristics:** Cross-sectional study. Neuropsychiatric patients, n = 155, consisting of a valid and invalid group. - **Location:** USA - **Result:** Cut-off score ≤ 40, sensitivity (SN) = 77%, specificity (SP) = 90%.   **Denning, 2012 (47)**   - **Study Characteristics of the 18 studies:** Range population, n = 6-604. Population sample: active military, mTBI litigants, pediatric neurology patients, forensic, toxic exposure, memory disordered, pain litigants, pain simulators, mixed clinical, psychogenic non-epileptic seizure, temporal lobe epilepsy, 80% disability, depressed inpatients, non-demented clinical, inpatient epilepsy. - **Location:** USA - **Result**: Weighted average across 18 studies that reported on SN and SP of TOMM, cut-off score ≤ 40, SN = 77%, SP = 92%.   **Bauer, 2007 (40)**   - **Study Characteristics:** Mild head-injury litigants, n = 105. - **Location:** USA - **Result:** Cut-off score ≤ 40, SN = 90%, SP = 89.3%. Cut-off score ≤ 35-40, negative predictive power (NPV) and positive predictive power (PPV) ≥ 80%, when base rate = 0.4.   **Teichner, 2004 (66)**   - **Study Characteristics:** Patients, n = 78, referred for a dementia examination due to complaints of memory loss. - **Location:** USA - **Result:** Overall correct classification rate = 94.7%.   **Tombaugh, 1997 (67)**   - **Study Characteristics:** Community-dwelling individuals, n = 475. Neurologically impaired patients (TBI, aphasia, cognitive impairment, and dementia), n = 161. - **Location:** Canada - **Result:** Criterion score 45, correct classification of non-dementia patients as not malingering = 95%. |
| Word Memory Test (WMT) | NA | **Bauer, 2007 (40)**   - **Study Characteristics:** Mild head-injury litigants, n = 105. - **Location:** USA - **Result:** Cut-off score ≤ 37, SN = 85.7%, SP = 81.8%. Cut-off score ≤ 31-35, negative predictive power (NPV) and positive predictive power (PPV) ≥ 80%, when base rate = 0.4.   **Green, 2003 (52)**   - **Study Characteristics:** Head injury patients (n = 535), neurological patients (strokes, aneurysms, multiple sclerosis, tumor, epilepsy, or other miscellaneous conditions; n = 89), major depression (n = 85), anxiety disorders (n = 18), orthopedic injuries (n = 77), chronic fatigue syndrome (n = 34), chronic pain syndrome or fibromyalgia (n = 61) and other conditions (n = 101). - **Location:** Canada - **Result:** Correlation between the effort measures (immediate recognition, delayed recognition, and consistency) of the WMT = 0.86-0.89 (high internal reliability). Correlation between the memory measures (multiple choice, paired associate, delayed free recall, and long delayed free recall) of the WMT = 0.71-0.90. Effort metrics of the WMT explained nearly 50% of the variance observed in 30,736 neuropsychological test results. SN = 96%, SP = 83%. |
| b Test | NA | **Roberson, 2013 (63)**   - **Study Characteristics:** Non-credible participants (n=212) and credible heterogenous neurophysiological participants (n=103). - **Location:** USA - **Result:** Cut-off score ≥ 155, SP = 99%, SN = 41%. Cut-off score of ≥ 82, SP ≥ 90%, SN = 68%.   **Marshall, 2010 (55)**   - **Study Characteristics:** 257 subjects referred for ADHD assessment. - **Location:** USA - **Result:** Cut-off score ≥120, SP = 97.5%, SN = 22%. Cut-off score ≥70, SP = 93%, SN = 47%. |
| Victoria Symptom Validity Test (VSVT) | NA | **Resch, 2021 (60)**   - **Study Characteristics:** Systematic review. Included studies: 17 validation studies (7 simulation based, 7 differential prevalence based, and 3 known group based). - **Result:** Optimal cut-off score ≤40, SN = 62%, SP = 88%. - The study advises to be careful with patients who have specific confirmed clinical conditions (e.g., dementia) and those with significant working memory deficits because of the potential for a higher risk of false positives. |
| Dot Counting Test (DCT) | NA | **Abramson, 2023 (37)**   - **Study Characteristics:** Population, n = 210, examined for ADHD using neuropsychological evaluation. - **Location:** USA - **Result:** Cut-off score ≥14, SN = 54.3%, SP = 92%.   **Rhoads, 2021 (62)**   - **Study Characteristics:** Cross-sectional study. Mixed, diverse neuropsychiatric population, n = 132. - **Location:** USA - **Result:** SN = 50%-67%, SP ≥ 89%.   **Rhoads, 2021 (61)**   - **Study Characteristics:** 157 patients administered for a neuropsychological evaluation. - **Location:** USA - **Result:** Cut-off score ≥15, SN = 59.5%, SP = 89.5%.   **Soble, 2018 (65)**   - **Study Characteristics:** Cross-sectional study. 77 veterans, who completed DCT for clinical evaluation. - **Location:** USA - **Result:** Classification accuracy = 83.1%. Cut-off score = 15, SN = 70%, SP = 88%.   **Bonello, 1997 (44)**   - **Study Characteristics:** 111 healthy American adults, mean age = 68.2 years. - **Location:** USA - **Result:** SP = 92.8%, Cronbach alpha = 0.41 |
| Medical Symptom Validity Test (MSVT) | NA | **Cerny, 2022 (45)**   - **Study Characteristics:** Cross-sectional data. Outpatients, n = 187, referred for neuropsychological evaluation. - **Location:** USA - **Result:** MSVT immediate recognition, SN = 60%, SP = 89%. MSVT delayed recognition, SN = 73%, SP = 86%. MSVT consensus, SN = 63%, SP = 89%.   **Resch, 2022 (59)**   - **Study Characteristics:** Cross-sectional data. Clinical patients n = 129, consisting of 2 groups; valid group (n = 98) and invalid group (n = 31). - **Location:** USA - **Result:** SN = 55% - 71%, SP = 91%-93%.   **Green, 2004 (51)**   - **Study Characteristics:** Subjects with clinical history of significant cognitive impairment. - **Location:** USA - **Result:** Cut-off score ≤85%, SN = 88%, SP = 91%. |
| **Criteria from Slick et al., Symptom Validity Tests (SVTs)** | | |
| Symptom Validity Scale (FBS)***** | Minnesota Multiphasic Personality Inventory-2 (MMPI-2) and Minnesota Multiphasic Personality Inventory-2 Restructured Form  (MMPI-2-RF) | **Armistead‑Jehle, 2022 (39)**   - **Study Characteristics:** Population, n = 1728 adult patients seen for neuropsychological evaluation in the author’s private practice over an 18-year period. - **Location:** USA - **Result:** Convergent validity FBS with Memory Complaints Inventory (MCI) = 0.53   **Jones, 2016 (54)**   - **Study Characteristics:** n = 300, consisting of 4 groups: non-malingering, probable malingering, probable-to-definite malingering, and definite malingering. - **Location:** USA - **Result:** Cut-off ≥25, SP ≥ 90%   **Peck, 2013 (58)**   - **Study Characteristics:** Sample (n = 45) consisting of valid TBI group, and invalid TBI group, and a psychogenic non-epileptic seizure group. - **Location:** USA - **Result:** Cut-off ≥27, false-positive classification rate = 7%.   **Tsushima, 2011 (68)**   - **Study Characteristics:** Sample (n = 281) consisting of mTBI litigants, and/or compensation seeking group. - **Location:** USA - **Result:** Cut-off ≥25, SP = 91%.   **Dionysus, 2011 (48)**   - **Study Characteristics:** Sample of head injured patients consisting of a probable negative response group (n = 37), and a presumed valid group (n = 42). - **Location:** USA - **Result:** Cut-off ≥25, SP ≥ 90%. |
| Symptom Validity Scale-Reconstructed (FBS-r) |  | **Gass, 2012 (49)**   - **Study Characteristics:** 303 neuropsychological referrals, non-forensic. - **Location:** USA - **Result:** Reliability (internal consistency) = 0.747 |
| Response Bias Scale (RBS) |  | **Armistead‑Jehle, 2022 (39)**   - **Study Characteristics:** Population, n = 1728 adult patients seen for neuropsychological evaluation in the author’s private practice over an 18-year period. - **Location:** USA - **Result:** Convergent validity of RBS with MCI = 0.69   **Dionysus, 2011 (48)**   - **Study Characteristics:** Sample of head injured patients consisting of a probable negative response group (n = 37), and a presumed valid group (n = 42). - **Location:** USA - **Result:** Cut-off ≥14-15, SP = 93%.   **Tsushima, 2011 (68)**   - **Study Characteristics:** Sample (n = 281) consisting of mTBI litigants, and/or compensation seeking group. - **Location:** USA - **Result:** Cut-off ≥13, SP ≥ 91%.   **Gervais, 2007 (50)**   - **Study Characteristics**: n = 1,212 (archival data), nonhead-injury disability claimants referred to private psychology practice (first authors’ private practice). - **Location:** USA - **Result:** Cut-off 17, SP = 95%, SN = 25%, Positive Predictive Power (PPP) = 0.77, Negative Predictive Power (NPP) = 0.66. |
| Infrequent Somatic Reponses (Fs) |  | **Armistead‑Jehle, 2022 (39)**   - **Study Characteristics:** Population, n = 1728 adult patients seen for neuropsychological evaluation in the author’s private practice over an 18-year period. - **Location:** USA - **Result:** Convergent validity of Fs with MCI = 0.54   **Whitman, 2022 (69)**   - **Study Characteristics:** Subjects, n = 378, administered for psychological evaluation. - **Location:** Puerto Rico - **Result:** Cronbach α = 0.64   **Jones, 2016 (54)**   - **Study Characteristics:** n = 300, consisting of 4 groups: non-malingering, probable malingering, probable-to-definite malingering, and definite malingering. - **Location:** USA - **Result:** Cut-off ≥6, SP = 94% |
| Henry-Heilbronner Index (HHI)/(HHI-r) |  | **Jones, 2016 (54)**   - **Study Characteristics:** Population, n = 300, consisting of 4 groups: non-malingering, probable malingering, probable-to-definite malingering, and definite malingering. - **Location:** USA - **Result:** Cut-off ≥9, SP ≥ 90%   **Henry, 2013 (53)**   - **Study Characteristics:** n = 119 adults, consisting of probable malingering and definite malingering groups. - **Location:** USA - **Result:** Cut-off ≥7, classification accuracy rate = 84%, SN = 68.9%, SP = 93.2%.   **Dionysus, 2011 (48)**   - **Study Characteristics:** Sample of head injured patients consisting of a probable negative response group (n = 37), and a presumed valid group (n = 42). - **Location:** USA - **Result:** Cut-off ≥12, SP = 93%. |
| Total Score | Structured Inventory of Malingered Symptomatology (SIMS) | **Lace, 2021 (70)**   - **Study Characteristics:** Population, n = 67, referred for neuropsychological evaluation. - **Location:** USA - **Result:** Cut-off ≥ 2, SP = 96%, SN = 53% |
| **Criteria from Bianchini et al**** | | |
| Portland Digital Recognition Test (PDRT) | NA | **Bianchini, 2001 (41)**   - **Study Characteristics:** TBI subjects, n = 151, referred for neuropsychological evaluation. - **Country:** USA - **Result**: SN = 77%, SP = 100%.   **Binder, 1993 (42)**   - **Study Characteristics:** Patients (n = 47) with TBI. - **Location:** USA - **Result:** Convergence validity of PDRT and WAIS-R Full Scale IQ = 0.49. In patients with intractable epilepsy, r = 0.55.   **Binder, 1991 (43)**   - **Study Characteristics:** Sample (n = 139) consisted of 6 groups of adults: nonpatient simulators, nonpatient controls, patients with affective disorders not seeking compensation, patients with brain dysfunction not seeking compensation, patients with well-documented brain dysfunction seeking financial compensation, and mild head trauma patients seeking compensation. - **Location:** USA - **Result:** Split-half reliability, for nonpatient controls = 0.92. Split-half reliability, for brain dysfunction patients = 0.82. |
| Reliable Digit Span (RDS) | NA | **Schroeder, 2012 (64)**   - **Study Characteristics:** Systematic review, included 20 validation studies, analyzing the SP and SN. - **Result:** Cut-off ≤7, SP = 82% (using weighted averages), 85% (using Bayesian method), SN = 48% (using weighted averages), 58% (using Bayesian method). |
| Meyers Index (MI) | Minnesota Multiphasic Personality Inventory-2 (MMPI-2) | **Meyers, 2002 (56)**   - **Study Characteristics:** Group 1 patients (n = 100), were not involved in litigation or disability proceedings (42 self-reported a mean loss of consciousness, 26 had history of a TBI, the rest had a variety of other conditions).   Group 2 patients (n = 100) were involved in litigation proceedings (49 self-reported loss of consciousness, 31 had TBI, and the rest had a variety of other conditions. Both groups claimed having chronic pain. MI is made up of 7 combined validity scales from the MMPI-2, for assessing malingering in chronic pain patients. The 7 scales are: F–K, FT, FBS, F(p), Ds-r, Es, O-S.   - **Location:** USA - **Result:** MI correctly classified 100% of non-litigants, with a cut-off 5, thus SP = 100%, SN = 86%. |
| Working Memory Index (WMI) | WAIS-III | **Ovsiew, 2020 (57)**   - **Study Characteristics:** Cross-sectional study. Mixed clinical sample, n = 227, who completed neuropsychological evaluations. - **Location:** USA - **Result:** Cut off ≤82, SN = 32.0%, SP = 88.2%, Base Rate = 0.4, positive predictive power = 64%, negative predictive power = 66%.   **Alloway, 2008 (38)**   - **Study Characteristics:** Sample consisted of a low working memory group, n = 28, and an average working memory group, n = 37. - **Result:** Test-retest reliability = 0.83. |
| Processing Speed Index (PSI) |  | **Ovsiew, 2020 (57)**   - **Study Characteristics:** Cross-sectional study. Mixed clinical sample, n = 227, who completed neuropsychological evaluations. - **Location:** USA - **Result:** Cut-off ≤78, SN = 28.0%, SP = 94.1%, at Base Rate = 0.4, positive predictive power = 0.76, negative predictive   power = 0.66. |

*****Fake Bad Scale (FBS) renamed to Symptom Validity Scale.

******TOMM and FBS are also included in the criteria by Bianchini et al.

NA: Not applicable
