# Supplementary material for: Prevalence of symptom exaggeration among North American independent medical evaluation examinees: A systematic review of observational studies
Source: PLoS One. 2025 Jun 25;20(6):e0324684. doi: 10.1371/journal.pone.0324684 (PMC12193048; doi:10.1371/journal.pone.0324684)
Supplement: S5 Table — (DOCX) [file pone.0324684.s005.docx]

**S5 Table:** Included and excluded studies at full text screening with reasons

|  | Author (Year) | Title | Reason for exclusion |
| --- | --- | --- | --- |
| Included studies at full text screening | | | |
| 1 | Lees-Haley, 1991 | A fake bad scale on the MMPI-2 for personal injury claimants | Not applicable |
| 2 | Greiffenstein, 1995 | MMPI-2 validity scales versus domain specific measures in detection of factitious traumatic brain injury | Not applicable |
| 3 | Suhr, 1997 | Memory performance after head injury: Contributions of malingering, litigation status, psychological factors, and medication use | Not applicable |
| 4 | Costa, 1999 | Psychiatric detection of exaggeration in reports of memory impairment. | Not applicable |
| 5 | Van Gorp, 1999 | How Well Do Standard Clinical Neuropsychological Tests Identify Malingering? A Preliminary Analysis* | Not applicable |
| 6 | Sweet, 2000 | Further Investigation of Traumatic Brain Injury Versus Insufficient Effort with the California Verbal Learning Test | Not applicable |
| 7 | Greve, 2003 | Detecting malingered performance on the Wechsler Adult Intelligence Scale Validation of Mittenberg's approach in traumatic brain injury | Not applicable |
| 8 | Lu, 2003 | Effectiveness of the Rey-Osterrieth Complex Figure Test and the Meyers and Meyers recognition trial in the detection of suspect effort | Not applicable |
| 9 | Barrash, 2004 | Detecting poor effort and malingering with an expanded version of the Auditory Verbal Learning Test (AVLTX): Validation with clinical samples | Not applicable |
| 10 | Heinly, 2005 | WAIS Digit Span-based indicators of malingered neurocognitive dysfunction: Classification accuracy in traumatic brain injury | Not applicable |
| 11 | Curtis, 2006 | California Verbal Learning Test indicators of malingered neurocognitive dysfunction: Sensitivity and specificity in traumatic brain injury | Not applicable |
| 12 | Etherton, 2006a | Pain, malingering and the WAIS-III working memory index | Not applicable |
| 13 | Greve, 2006a | Sensitivity and specificity of MMPI-2 validity scales and indicators to malingered neurocognitive dysfunction in traumatic brain injury | Not applicable |
| 14 | Greve, 2006b | Classification accuracy of the Test of Memory Malingering in traumatic brain injury: Results of a known-groups analysis | Not applicable |
| 15 | Greve, 2006c | Classification accuracy of the Portland Digit Recognition Test in traumatic brain injury: Results of a known-groups analysis | Not applicable |
| 16 | Greve, 2006d | The prevalence of cognitive malingering in persons reporting exposure to occupational and environmental substances | Not applicable |
| 17 | Ardolf, 2007 | Base rates of negative response bias and malingered neurocognitive dysfunction among criminal defendants referred for neuropsychological evaluation | Not applicable |
| 18 | Greve, 2007 | The Booklet Category Test and malingering in traumatic brain injury: Classification accuracy in known groups | Not applicable |
| 19 | Henry, 2007 | Probable malingering and performance on the Continuous Visual Memory Test | Not applicable |
| 20 | O’Bryant, 2007 | Test of memory malingering (TOMM) trial 1 as a screening measure for insufficient effort | Not applicable |
| 21 | Greve, 2007a | Malingering in toxic exposure: Classification accuracy of Reliable Digit Span and WAIS-III Digit Span scaled scores | Not applicable |
| 22 | Aguerrevere, 2008 | Detecting malingering in traumatic brain injury and chronic pain with an abbreviated version of the Meyers Index for the MMPI-2. Archives of Clinical Neuropsychology | Not applicable |
| 23 | Curtis, 2008 | Verbal fluency indicators of malingering in traumatic brain injury: Classification accuracy in known groups | Not applicable |
| 24 | Greve, 2008 | Observed versus estimated IQ as an index of malingering in traumatic brain injury: Classification accuracy in known groups | Not applicable |
| 25 | Ord, 2008 | Using the Wechsler Memory Scale-III to detect malingering in mild traumatic brain injury | Not applicable |
| 26 | Greve, 2008b | Detecting malingering in traumatic brain injury and chronic pain: A comparison of three forced-choice symptom validity tests | Not applicable |
| 27 | Henry, 2009 | Comparison of the MMPI-2 restructured demoralization scale, depression scale, and malingered mood disorder scale in identifying non-credible symptom reporting in personal injury litigants and disability claimants | Not applicable |
| 28 | Greve, 2009 | Malingering detection with the Wisconsin Card Sorting Test in mild traumatic brain injury | Not applicable |
| 29 | Greve, 2009a | Detecting malingered pain-related disability: Classification accuracy of the Portland Digit Recognition Test | Not applicable |
| 30 | Greve, 2009b | Are the original and second edition of the California Verbal Learning Test equally accurate in detecting malingering? | Not applicable |
| 31 | Greve, 2009c | Detecting malingered pain-related disability: Classification accuracy of the Test of Memory Malingering | Not applicable |
| 32 | Greve, 2009d | Prevalence of malingering in patients with chronic pain referred for psychologic evaluation in a medico-legal context | Not applicable |
| 33 | Bortnik, 2010 | Examination of various WMS-III logical memory scores in the assessment of response bias | Not applicable |
| 34 | Curtis, 2010 | Criterion groups validation of the Seashore Rhythm Test and Speech Sounds Perception Test for the detection of malingering in traumatic brain injury | Not applicable |
| 35 | Greve, 2010 | The Reliable Digit Span test in chronic pain: Classification accuracy in detecting malingered pain-related disability | Not applicable |
| 36 | Ord, 2010 | Detection of malingering in mild traumatic brain injury with the Conners' Continuous Performance Test–II | Not applicable |
| 37 | Aguerrevere, 2011 | Classification accuracy of the Millon Clinical Multiaxial Inventory–III modifier indices in the detection of malingering in traumatic brain injury | Not applicable |
| 38 | Roberson, 2013 | Cross validation of the b Test in a large known groups sample | Not applicable |
| 39 | Bianchini, 2014 | Accuracy of the Modified Somatic Perception Questionnaire and Pain Disability Index in the detection of malingered pain-related disability in chronic pain | Not applicable |
| 40 | Guise, 2014 | Assessment of performance validity in the S troop Color and Word Test in mild traumatic brain injury patients: A criterion‐groups validation design | Not applicable |
| 41 | Patrick, 2014 | Psychological characteristics of individuals who put forth inadequate cognitive effort in a secondary gain context | Not applicable |
| 42 | Aguerrevere, 2017 | Clusters of financially incentivized chronic pain patients using the Minnesota Multiphasic Personality Inventory-2 Restructured Form (MMPI-2-RF) | Not applicable |
| 43 | Bianchini, 2018 | Classification accuracy of the Minnesota Multiphasic Personality Inventory-2 (MMPI-2)-Restructured form validity scales in detecting malingered pain-related disability | Not applicable |
| 44 | Curtis, 2019 | Detecting malingered pain-related disability with the pain catastrophizing scale: a criterion groups validation study | Not applicable |
| Excluded studies at full text screening | | | |
| 1 | Gorman, 1982 | Defining malingering | Not an observational study |
| 2 | Brandt 1985 | Uncovering malingered amnesia | Not an observational study |
| 3 | Lande, 1989 | Malingering | Not an observational study |
| 4 | Franzen, 1990 | The detection of malingering in neuropsychological assessment | Not an observational study |
| 5 | Perry, 1990 | The susceptibility of the Rorschach to malingering: A critical review | Not an observational study |
| 6 | Rogers, 1990 | Development of a new classificatory model of malingering | Not an observational study |
| 7 | Berry, 1991 | Detection of malingering on the MMPI: A meta-analysis | Not an observational study |
| 8 | Resnick, 1993 | Defrocking the fraud: the detection of malingering. | Not an observational study |
| 9 | Nies, 1994 | Neuropsychological assessment and malingering: A critical review of past and present strategies | Not an observational study |
| 10 | Hanes, 1995 | Detecting the malingering of cognitive deficits: An update | Not an observational study |
| 11 | Zinn, 1996 | Physician Perspectives on the Ethical Aspects of Disability Determination | Not an observational study |
| 12 | LoPiccolo, 1999 | Current issues in the diagnosis and management of malingering | Not an observational study |
| 13 | Slick, 1999 | Diagnostic Criteria for Malingered Neurocognitive Dysfunction: Proposed Standards for Clinical Practice and Research* | Not an observational study |
| 14 | Youngjohn, 1999 | Comment: Warning malingerers produces more sophisticated malingering | Not an observational study |
| 15 | Irby, 2000 | Detecting malingering in a clinical sample with the Wechsler memory scale-revised | Not an observational study |
| 16 | Millis, 2001 | Assessment of response bias in mild head injury: Beyond malingering tests | Not an observational study |
| 17 | Ameis, 2002 | The independent medical examination | Not an observational study |
| 18 | Langeluddecke, 2003 | Quantitative measures of memory malingering on the Wechsler Memory Scale—Third edition in mild head injury litigants | Not an observational study |
| 19 | Wessely, 2003 | Malingering: historical perspectives | Not an observational study |
| 20 | Mendelson, 2004 | Malingering pain in the medicolegal context | Not an observational study |
| 21 | Bianchini, 2005 | On the diagnosis of malingered pain-related disability: Lessons from cognitive malingering research | Not an observational study |
| 22 | Conroy, 2006 | The definition of malingering | Not an observational study |
| 23 | Horwitz, 2006 | A review of internet sites regarding independent medical examinations: Implications for clinical neuropsychological practitioners | Not an observational study |
| 24 | Iverson, 2006 | Ethical issues associated with the assessment of exaggeration, poor effort, and malingering | Not an observational study |
| 25 | Sisung, 2006 | Predictive utility of the MMPI-2 fake bad scale on Wisconsin card sorting test performance during forensic traumatic brain evaluations | Not an observational study |
| 26 | Aronoff, 2007 | Evaluating malingering in contested injury or illness | Not an observational study |
| 27 | McDermott, 2007 | Malingering in the medical setting | Not an observational study |
| 28 | Suhr, 2007 | Coaching and malingering: A review | Not an observational study |
| 29 | Haberstroh, 2008 | IE (IME) Excesses and how to encounter them | Not an observational study |
| 30 | Mitchell 2008 | Relationship between the performance on the MMPI-2 fake bad scaled and the word memory subtests in the detection of malingering during forensic brain trauma evaluations | Not an observational study |
| 31 | Taiwo, 2008 | Impairment and Disability Evaluation: The Role of the Family Physician | Not an observational study |
| 32 | Dewa, 2009 | Cost, Effectiveness, and Cost-Effectiveness of a Collaborative Mental Health Care Program for People Receiving Short-Term Disability Benefits for Psychiatric Disorders | Not an observational study |
| 33 | Edwards, 2009 | Disability Guideline, Role Conflicts, and Treatment Records | Not an observational study |
| 34 | Hameed, 2009 | Independent Medical Examinations: Facts and Fallacies | Not an observational study |
| 35 | Jahn, 2009 | The lines are less blurred: independent medical examinations and limited doctor-patient relationship | Not an observational study |
| 36 | Latner, 2009 | Independent exam seems to blur roles | Not an observational study |
| 37 | Gutheil, 2010 | Commentary: Tarasoff Duties Arising from a Forensic Independent Medical Examination | Not an observational study |
| 38 | Skakic, 2010 | Work disability among adult psychiatric patients | Not an observational study |
| 39 | William, 2010 | Disability and Occupational Assessment: Objective Diagnosis and Quantitative Impairment Rating | Not an observational study |
| 40 | Cupon, 2011 | Independent medical examinations and depression | Not an observational study |
| 41 | Duhamel, 2011 | Detection of malingering and somatization using the Pain Symptom Ratings, 2nd Edition | Not an observational study |
| 42 | Jasinski, 2011 | Use of the Wechsler Adult Intelligence Scale Digit Span subtest for malingering detection: A meta-analytic review | Not an observational study |
| 43 | Silver, 2012 | Effort, exaggeration and malingering after concussion | Not an observational study |
| 44 | Bass, 2014 | Factitious disorders and malingering: challenges for clinical assessment and management | Not an observational study |
| 45 | Amlani, 2016 | Malingering by proxy: A literature review and current perspectives | Not an observational study |
| 46 | Bashem, 2017 | Performance validity assessment of bona fide and malingered traumatic brain injury using novel eye-tracking systems | Not an observational study |
| 47 | Berry, 2017 | Feigning issues in brain injury | Not an observational study |
| 48 | Bianchini, 2017 | The financial incentive effect: It's not just malingering | Not an observational study |
| 49 | Borchman, 2017 | Examining the role of effort in embedded and self-report measures in the neuropsychological testing of traumatic brain injury | Not an observational study |
| 50 | Geba, 2017 | Assessment of malingered neurocognitive impairment: Examining the use of embedded measures in place of symptom validity tests | Not an observational study |
| 51 | Hampton, 2017 | Psychopathy and malingering: Examining proneness to malinger in an inmate sample | Not an observational study |
| 52 | Nielsen, 2017 | Examining the relationship between depression and malingering in traumatic brain injury evaluations in a military population | Not an observational study |
| 53 | Nijdam-Jones 2017 | Cross-cultural feigning assessment: A systematic review of feigning instruments used with linguistically, ethnically, and culturally diverse samples | Not an observational study |
| 54 | Ramachandran, 2017 | Identification of stimulant misuse and malingering of symptoms of attention deficit hyperactivity disorder | Not an observational study |
| 55 | Rowland, 2017 | 2017 American Neuropsychiatric Association Annual Meeting Abstracts | Not an observational study |
| 56 | Silk-Eglit, 2017 | Reduced Conscious Recollection and its Detection in Three Performance Validity Tests: A Dual Task Interference Investigation | Not an observational study |
| 57 | Suhr, 2017 | The Importance of Assessing for Validity of Symptom Report and  Performance in Attention Deficit/Hyperactivity Disorder (ADHD): Introduction to the Special Section on Noncredible Presentation in ADHD | Not an observational study |
| 58 | Tracy, 2017 | Malingering mental disorders: clinical assessment | Not an observational study |
| 59 | Young, 2017 | PTSD in Court III: Malingering, assessment, and the law | Not an observational study |
| 60 | Bender, 2018 | Neuropsychological models of feigned cognitive deficits | Not an observational study |
| 61 | Bender, 2018 | Malingered traumatic brain injury | Not an observational study |
| 62 | Frederick, 2018 | Feigned amnesia and memory problems | Not an observational study |
| 63 | Garcia-Willingham, 2018 | Assessment of feigned cognitive impairment using standard neuropsychological tests | Not an observational study |
| 64 | Granacher, 2018 | Feigned medical presentations | Not an observational study |
| 65 | Merten, 2018 | False symptom claims and symptom validity assessment | Not an observational study |
| 66 | Rogers, 2018 | Detection strategies for malingering and defensiveness | Not an observational study |
| 67 | Rogers, 2018 | Clinical assessment of malingering and deception | Not an observational study |
| 68 | Smith, 2018 | Brief measures for the detection of feigning and impression management | Not an observational study |
| 69 | Tylicki, 2018 | Correction to comparability of Structured Interview of Reported Symptoms (SIRS) and Structured Interview of Reported Symptoms–Second Edition (SIRS-2) Classifications with External Response Bias Criteria, | Not an observational study |
| 70 | Walczyk, 2018 | A review of approaches to detecting malingering in forensic contexts and promising cognitive load-inducing lie detection techniques | Not an observational study |
| 71 | Yilmaz, 2018 | Field validity of a measure of malingering with motor-vehicle accident claimants | Not an observational study |
| 72 | An, 2019 | Performance Validity Testing for Individuals with Limited English Proficiency | Not an observational study |
| 73 | Armstrong, 2019 | Disability assessment | Not an observational study |
| 74 | Bass, 2019 | Malingering and factitious disorder | Not an observational study |
| 75 | Barkley, 2019 | Neuropsychological testing is not useful in the diagnosis of ADHD: Stop it (or prove it) | Not an observational study |
| 76 | Ernst, 2019 | Detecting adult attention-deficit/hyperactivity disorder malingering using the behavior rating inventory of executive functioning-adult | Not an observational study |
| 77 | Golanics, 2019 | Malingering undetected successfully: Does extrinsic motivation and coaching have a significant impact? | Not an observational study |
| 78 | Grossi, 2019 | Assessing feigning with the malingering assessment of psychopathology (map) in a forensic psychiatric sample | Not an observational study |
| 79 | Lemeunier, 2019 | Reliability and validity of self-reported questionnaires to measure pain and disability in adults with neck pain and its associated disorders: part 3-a systematic review from the CADRE Collaboration | Not an observational study |
| 80 | McBride, 2019 | Latent class analysis of malingering classifications using performance and symptom validity measures in a civil forensic setting | Not an observational study |
| 81 | Seybert-Williams, 2019 | The effectiveness of brief measures in screening for malingered chronic pain in a primary care setting | Not an observational study |
| 82 | Saltychev, 2019 | Psychometric properties of 12-item self-administered World Health Organization disability assessment schedule 2.0 (WHODAS 2.0) among general population and people with non-acute physical causes of disability - systematic review | Not an observational study |
| 83 | Wallace, 2019 | A meta-analysis of malingering detection measures for attention-deficit/hyperactivity disorder | Not an observational study |
| 84 | Zasler, 2019 | Validity Assessment in Traumatic Brain Injury Impairment and Disability Evaluations | Not an observational study |
| 85 | Clark, 2020 | A Systematic Review and Meta-Analysis of the Utility of the Test of Memory Malingering in Pediatric Examinees | Not an observational study |
| 86 | Kosky, 2020 | Methods of detection of feigned attention deficit hyperactivity disorder in a college-student population | Not an observational study |
| 87 | Lace, 2020 | Detecting feigned neurocognitive impairment related to mild traumatic brain injury: Comparing embedded effort indicators to standalone measures | Not an observational study |
| 88 | Liberti, 2020 | Symptom Validity Testing | Not an observational study |
| 89 | Luque-Suarez, 2020 | Is kinesiophobia and pain catastrophising at baseline associated with chronic pain and disability in whiplash-associated disorders? A systematic review | Not an observational study |
| 90 | Malm, 2020 | Detecting Feigning in Adolescents on the Personality Assessment Inventory— Adolescent Form | Not an observational study |
| 91 | Martin, 2020 | A systematic review and meta-analysis of the Test of Memory Malingering in adults: Two decades of deception detection | Not an observational study |
| 92 | Regan, 2020 | Malingered cognitive symptoms in head injury | Not an observational study |
| 93 | Sahoo, 2020 | Concepts and controversies of malingering: A re-look | Not an observational study |
| 94 | Sherman, 2020 | Multidimensional malingering criteria for neuropsychological assessment: A 20-year update of the malingered neuropsychological dysfunction criteria | Not an observational study |
| 95 | Siegel, 2020 | The effects of incentivizing ADHD related non-credible responding on neuropsychological measures and performance validity tests | Not an observational study |
| 96 | Silva, 2020 | Identification of categories of the International Classification of Functioning, Disability and Health in functional assessment measures for stroke survivors: a systematic review | Not an observational study |
| 97 | Suh, 2020 | Utility of the Response Bias Scale in a Diverse Clinical Sample | Not an observational study |
| 98 | Wygant, 2020 | Assessment of noncredible reporting and responding | Not an observational study |
| 99 | Clayton, 2021 | Malingering Detection among Accommodation-Seeking University Students | Not an observational study |
| 100 | Monaro, 2021 | The detection of malingering in whiplash-related injuries: a targeted literature review of the available strategies | Not an observational study |
| 101 | Bellman, 2022 | Malingering of psychotic symptoms in psychiatric settings: theoretical aspects and clinical considerations | Not an observational study |
| 102 | Leonhard, 2023 | Review of statistical and methodological issues in the forensic prediction of malingering from validity tests: Part I: Statistical issues | Not an observational study |
| 103 | Leonhard, 2023 | Review of statistical and methodological issues in the forensic prediction of malingering from validity tests: Part II—Methodological issues | Not an observational study |
|  | | | |
| 1 | Lees-Haley, 1991 | MMPI-2 F and F-K scores of personal injury malingerers in vocational neuropsychological and emotional distress claims | All groups were pre-selected for exaggeration status. The study purpose was to test an instrument to pick them up, not to establish the prevalence of symptom exaggeration. |
| 2 | Prigatano, 1993 | Digit memory test: Unequivocal cerebral dysfunction and suspected malingering | All groups were pre-selected for exaggeration status. The study purpose was to test an instrument to pick them up, not to establish the prevalence of symptom exaggeration. |
| 3 | Greiffenstein, 1994 | Validation of Malingered Amnesia Measures with a Large Clinical Sample | All groups were pre-selected for exaggeration status. The study purpose was to test an instrument to pick them up, not to establish the prevalence of symptom exaggeration. |
| 4 | Millis, 1995 | The California verbal learning test in the detection of incomplete effort in neuropsychological evaluation | All groups were pre-selected for exaggeration status. The study purpose was to test an instrument to pick them up, not to establish the prevalence of symptom exaggeration. |
| 5 | Viglione, 1995 | Maximizing Internal and External Validity in MMPI Malingering Research: A Study of a Military Population | All groups were pre-selected for exaggeration status. The study purpose was to test an instrument to pick them up, not to establish the prevalence of symptom exaggeration. |
| 6 | Ganellen, 1996 | Can Psychosis Be Malingered on the Rorschach? An Empirical Study | All groups were pre-selected for exaggeration status. The study purpose was to test an instrument to pick them up, not to establish the prevalence of symptom exaggeration. |
| 7 | Smith, 1996 | Compensation seeking, comorbidity and apparent exaggeration of PTSD symptoms among Vietnam combat veterans | All groups were pre-selected for exaggeration status. The study purpose was to test an instrument to pick them up, not to establish the prevalence of symptom exaggeration. |
| 8 | Slick, 1996 | Victoria Symptom Validity Test: Efficiency for Detecting Feigned Memory Impairment and Relationship to Neuropsy- chological Tests and MMPI-2 Validity Scales | All groups were pre-selected for exaggeration status. The study purpose was to test an instrument to pick them up, not to establish the prevalence of symptom exaggeration. |
| 9 | Larrabee, 1998 | Somatic Malingering on the MMPI and MMPI-2 in Personal Injury Litigants* | All groups were pre-selected for exaggeration status. The study purpose was to test an instrument to pick them up, not to establish the prevalence of symptom exaggeration. |
| 10 | Millis, 1998 | Detection of Incomplete Effort on the Wechsler Adult Intelligence Scale-Revised: A Cross-Validation | All groups were pre-selected for exaggeration status. The study purpose was to test an instrument to pick them up, not to establish the prevalence of symptom exaggeration. |
| 11 | Meyers, 2000 | Assessment of Malingering in Chronic Pain Patients Using Neuropsychological Tests | All groups were pre-selected for exaggeration status. The study purpose was to test an instrument to pick them up, not to establish the prevalence of symptom exaggeration. |
| 12 | Sbordone, 2000 | The Use of Significant Others to Enhance the Detection of Malingerers From Traumatically Brain-Injured Patients | All groups were pre-selected for exaggeration status. The study purpose was to test an instrument to pick them up, not to establish the prevalence of symptom exaggeration. |
| 13 | Greiffenstein, 2002 | The Fake Bad Scale in Atypical and Severe Closed Head Injury Litigants | All groups were pre-selected for exaggeration status. The study purpose was to test an instrument to pick them up, not to establish the prevalence of symptom exaggeration. |
| 14 | Iverson, 2002 | Detecting Exaggeration and Malingering With the Trail Making Test | All groups were pre-selected for exaggeration status. The study purpose was to test an instrument to pick them up, not to establish the prevalence of symptom exaggeration. |
| 15 | Mathias, 2002 | Detecting Malingered Neurocognitive Dysfunction Using the Reliable Digit Span in Traumatic Brain Injury | All groups were pre-selected for exaggeration status. The study purpose was to test an instrument to pick them up, not to establish the prevalence of symptom exaggeration. |
| 16 | Larrabee, 2003 | Detection of Malingering Using Atypical Performance Patterns on Standard Neuropsychological Tests | All groups were pre-selected for exaggeration status. The study purpose was to test an instrument to pick them up, not to establish the prevalence of symptom exaggeration. |
| 17 | Greiffenstein, 2004 | The Fake Bad Scale and MMPI-2 F-Family in Detection of Implausible Psychological Trauma Claims | All groups were pre-selected for exaggeration status. The study purpose was to test an instrument to pick them up, not to establish the prevalence of symptom exaggeration. |
| 18 | Langeluddecke, 2004 | Validation of the Rarely Missed Index (RMI) in Detecting Memory Malingering in Mild Head Injury Litigants | All groups were pre-selected for exaggeration status. The study purpose was to test an instrument to pick them up, not to establish the prevalence of symptom exaggeration. |
| 19 | Etherton, 2005 | Sensitivity and Specificity of Reliable Digit Span in Malingered Pain-Related Disability | All groups were pre-selected for exaggeration status. The study purpose was to test an instrument to pick them up, not to establish the prevalence of symptom exaggeration. |
| 20 | Greve, 2006e | Classification accuracy of the Test of Memory Malingering in persons reporting exposure to environmental and industrial toxins: Results of a known-groups analysis | All groups were pre-selected for exaggeration status. The study purpose was to test an instrument to pick them up, not to establish the prevalence of symptom exaggeration. |
| 21 | Haber, 2006 | Replication of the test of memory malingering (TOMM) in a traumatic brain injury and head trauma sample | All groups were pre-selected for exaggeration status. The study purpose was to test an instrument to pick them up, not to establish the prevalence of symptom exaggeration. |
| 22 | Nitch, 2006 | The utility of the rey word recognition test in the detection of suspect effort | All groups were pre-selected for exaggeration status. The study purpose was to test an instrument to pick them up, not to establish the prevalence of symptom exaggeration. |
| 23 | Ross, 2006 | Detecting insufficient effort using the seashore rhythm and speech-sounds perception tests in head injury | All groups were pre-selected for exaggeration status. The study purpose was to test an instrument to pick them up, not to establish the prevalence of symptom exaggeration. |
| 24 | Flaro, 2007 | Word Memory Test failure 23 times higher in mild brain injury than in parents seeking custody: The power of external incentives | All groups were pre-selected for exaggeration status. The study purpose was to test an instrument to pick them up, not to establish the prevalence of symptom exaggeration. |
| 25 | Alwes, 2008 | Screening for feigning in a civil forensic setting | All groups were pre-selected for exaggeration status. The study purpose was to test an instrument to pick them up, not to establish the prevalence of symptom exaggeration. |
| 26 | Curtis, 2009 | The Wechsler Adult Intelligence Scale–III and Malingering in Traumatic Brain Injury | All groups were pre-selected for exaggeration status. The study purpose was to test an instrument to pick them up, not to establish the prevalence of symptom exaggeration. |
| 27 | Kim, 2010 | Sensitivity and Specificity of a Digit Symbol Recognition Trial in the Identification of Response Bias | All groups were pre-selected for exaggeration status. The study purpose was to test an instrument to pick them up, not to establish the prevalence of symptom exaggeration. |
| 28 | Strutt, 2012 | Assessing sub-optimal performance with the Test of Memory Malingering in Spanish speaking patients with TBI | All groups were pre-selected for exaggeration status. The study purpose was to test an instrument to pick them up, not to establish the prevalence of symptom exaggeration. |
| 29 | Chafetz, 2013 | Feigning a Severe Impairment Profile | All groups were pre-selected for exaggeration status. The study purpose was to test an instrument to pick them up, not to establish the prevalence of symptom exaggeration. |
| 30 | Henry, 2013 | Use of the Color Trails Test as an Embedded Measure of Performance Validity | All groups were pre-selected for exaggeration status. The study purpose was to test an instrument to pick them up, not to establish the prevalence of symptom exaggeration. |
| 31 | Henry, 2013 | Derivation of the MMPI-2-RF Henry- Heilbronner Index-r (HHI-r) Scale | All groups were pre-selected for exaggeration status. The study purpose was to test an instrument to pick them up, not to establish the prevalence of symptom exaggeration. |
| 32 | Jones, 2013 | Life Outcomes of Anterior Temporal Lobectomy: Serial Long- term Follow-up Evaluations | All groups were pre-selected for exaggeration status. The study purpose was to test an instrument to pick them up, not to establish the prevalence of symptom exaggeration. |
| 33 | Reedy, 2013 | Cross validation of the Lu and colleagues (2003) Rey-Osterrieth Complex Figure Test effort equation in a large known-group sample | All groups were pre-selected for exaggeration status. The study purpose was to test an instrument to pick them up, not to establish the prevalence of symptom exaggeration. |
| 34 | Smith, 2014 | Compensation seeking, comorbidity and apparent exaggeration of PTSD symptoms among Vietnam combat veterans | All groups were pre-selected for exaggeration status. The study purpose was to test an instrument to pick them up, not to establish the prevalence of symptom exaggeration. |
|  | | | |
| 1 | Clark, 1988 | Back impairment and disability determination another attempt at objective, reliable rating | Not known group design |
| 2 | Binder, 1991 | Assessment of motivation after financially compensable minor head trauma | Not known group design |
| 3 | Lees-Haley, 1992 | Efficacy of MMPI-2 validity scales and MCMI_II modifier scales for detecting spurious PYSD claims: F, F-K, fake bad scale, ego strength, subtle obvious subscales, DIS and DEB | Not known group design |
| 4 | Millis, 1992 | The recognition memory test in  the detection of malingered and  exaggerated memory deficits | Not known group design |
| 5 | Tsushima, 1992 | Comparison of legal and medical referrals to neuropsychological examination following head injury | Not known group design |
| 6 | Binder, 1993a | Assessment of malingering after mild  head trauma with the portland digit  recognition test | Not known group design |
| 7 | Binder, 1993b | The Rey AVLT Recognition Memory Task Measures Motivational Impairment After Mild Head Trauma | Not known group design |
| 8 | Stutts, 1993 | Disability: A New Psychosocial perspective | Not known group design |
| 9 | Trueblood, 1993 | Malingering and other validity  considerations in the neuropsychological  evaluation of mild head injury | Not known group design |
| 10 | Millis, 1994 | Assessment of motivation and memory with the recognition memory test after financially compensable mild head injury | Not known group design |
| 11 | Okpaku, 1994 | Disability determinations for adults with mental disorders: social security administration vs independent judgements | Not known group design |
| 12 | Trueblood, 1994 | Qualitative and Quantitative Characteristics of Malingered and Other Invalid WAIS-R and Clinical Memory Data* | Not known group design |
| 13 | Boon, 1995 | Rey 15-item memorization and dot counting scores in a “stress” claim worker’s compensation population: relationship to personally (MCMI) scores | Not known group design |
| 14 | Rickards, 1995 |  | Not known group design |
| 15 | Earman, 1996 | Factors Influencing the Cost of Chronic Low Back Injuries: An Analysis of Data from Independent Medical Examinations | Not known group design |
| 16 | Greiffenstein, 1996 | Comparison of Multiple Scoring Methods for Rey's Malingered Amnesia Measures | Not known group design |
| 17 | Smith, 1996 |  | Not known group design |
| 18 | Lees-Haley, 1997 | MMPI-2 Base Rates for 492 Personal Injury Plaintiffs: Implications and Challenges for Forensic Assessment | Not known group design |
| 19 | Reitan, 1997 | Consistency of Neuropsychological Test Scores of Head- Injured Subjects Involved in Litigation Compared with Head- Injured Subjects Not Involved in Litigation: Development of the Retest Consistency Index* | Not known group design |
| 20 | Kay, 1998 | Pain clinic management of medico-legal litigants | Not known group design |
| 21 | Morel, 1998 | Development and preliminary validation of a forced-choice test of response bias for posttraumatic stress disorder | Not known group design |
| 22 | Meyers, 1998 | Validation of Reliable Digits for Detection of Malingering | Not known group design |
| 23 | Doss, 1999 | Victoria Symptom Validity Test: Compensation-Seeking vs. Non-Compensation-Seeking Patients in a General Clinical Setting | Not known group design |
| 24 | Green, 1999 | Detecting malingering in head injury litigation with the Word Memory Test | Not known group design |
| 25 | Gold, 1999 | Compensation seeking and extreme exaggeration of psychopathology among combat veterans evaluated for post-traumatic stress disorder | Not known group design |
| 26 | Meyers, 1999 | Detection of Malingerers Using the Rey Complex Figure and Recognition Trial | Not known group design |
| 27 | Williams, 1999 |  | Not known group design |
| 28 | Grote, 2000 | Performance of Compensation Seeking and Non- Compensation Seeking Samples on the Victoria Symptom Validity Test: Cross-validation and Extension of a Standardization Study | Not known group design |
| 29 | Slick, 2000 | California Verbal Learning Test Indicators of Suboptimal Performance in a Sample of Head-Injury Litigants | Not known group design |
| 30 | Suchy, 2000 | Information/Orientation Subtest of the Wechsler Memory Scale-Revised as an Indicator of Suspicion of Insufficient Effort* | Not known group design |
| 31 | Bianchini, 2001 | Classification Accuracy of the Portland Digit Recognition Test in Traumatic Brain Injury | Not known group design |
| 32 | Demakis, 2001 | Discrepancy between predicted and obtained QAIS-R IQ scores discriminates between traumatic brain injury and insufficient effort | Not known group design |
| 33 | Gervais, 2001a | Effects of coaching on symptom validity testing in chronic pain patients presenting for disability assessments | Not known group design |
| 34 | Gervais, 2001b | Effort testing in patients with fibromyalgia and disability incentives. | Not known group design |
| 35 | Green, 2001 | Effort has a greater effect on test scores than severe brain injury in compensation claimants | Not known group design |
| 36 | Green, 2001b | Effects of injury severity and cognitive exaggeration on olfactory deficits in head injury compensation claims | Not known group design |
| 37 | Green, 2001c | Validation of the Computerized Assessment of Response Bias in Litigating Patients with Head Injuries | Not known group design |
| 38 | Iverson, 2001 | Cognitive Complaints in Litigating Patients with Head Injuries or Chronic Pain | Not known group design |
| 39 | Iverson, 2001 | Can malingering be identified with the judgement of line orientation test? | Not known group design |
| 40 | Mailis, 2001 | Unexplainable Nondermatomal Somatosensory Deficits in Patients with Chronic Nonmalignant Pain in the Context of Litigation/Compensation: a Role for Involvement of Central Factors? | Not known group design |
| 41 | Mittenberg, 2001 | Identification of Malingered Head Injury on the Wechsler Adult Intelligence Scale ± 3rd Edition | Not known group design |
| 42 | Tsushima, 2001 | Comparison of the fake bad scale and other MMPI-2 validity scales with personal injury litigants | Not known group design |
| 43 | Bury, 2002 | The Detection of Feigned Uncoached and Coached Posttraumatic Stress Disorder With the MMPI–2 in a Sample of Workplace Accident Victims | Not known group design |
| 44 | Greve, 2002 | Using the Wisconsin Card Sorting Test to Detect Malingering: An Analysis of the Specificity of Two Methods in Non malingering Normal and Patient Samples | Not known group design |
| 45 | Mittenberg, 2002 | Base Rates of Malingering and Symptom Exaggeration | Not known group design |
| 46 | Rohling, 2002 | Depressive symptoms and neurocognitive test scores in patients passing symptom validity tests | Not known group design |
| 47 | Rohling, 2002a | Who is exaggerating cognitive impairment and who is not | Not known group design |
| 48 | Binder, 2003 | Motivation and Neuropsychological  Test Performance Following Mild Head  Injury | Not known group design |
| 49 | Larrabee 2003c | Exaggerated Pain Report in Litigants with Malingered Neurocognitive Dysfunction | Not known group design |
| 50 | Larrabee, 2003 |  | Not known group design |
| 51 | O'Bryant, 2003 |  | Not known group design |
| 52 | Temple, 2003 | Personality Characteristics of Patients Showing Suboptimal Cognitive Effort | Not known group design |
| 53 | Ross 2003 | MMPI-2 indices of psychological disturbance and attention and memory test performance in head injury | Not known group design |
| 54 | Williamson, 2003 | Evaluating Effort with the Word Memory Test and Category Test– Or Not: Inconsistencies in a Compensation-Seeking Sample | Not known group design |
| 55 | Backhaus, 2004 | Detection of Sub-Optimal Performance Using a Floor Effect Strategy in Patients with Traumatic Brain Injury | Not known group design |
| 56 | Gervais, 2004 | A comparison of WMT, CARB, and TOMM failure rates in non-head injury disability claimants | Not known group design |
| 57 | Ross 2004 | Detecting Incomplete Effort on the  MMPI-2: An Examination of the Fake-  Bad Scale in Mild Head Injury | Not known group design |
| 58 | Lax, 2004 | Medical evaluation of work- related illness: Evaluations by a treating occupational medicine specialist and by independent medical examiners compared | Not known group design |
| 59 | Moore, 2004 | Predictors of invalid neuropsychological test performance after traumatic brain injury | Not known group design |
| 60 | O'Bryant, 2004 |  | Not known group design |
| 61 | Slick, 2004 | Detecting malingering: a survey of experts’ practices | Not known group design |
| 62 | Constantinou, 2005 | Is poor performance on recognition memory effort measures indicative of generalized poor performance on neuropsychological tests? | Not known group design |
| 63 | Mallinson, 2005 | A New Set of Criteria for Evaluating Malingering in Work-Related Vestibular Injury | Not known group design |
| 64 | Greve, 2006 | Should the Retention trial of the Test of Memory Malingering be optional? | Not known group design |
| 65 | Lange, 2006 | Suppressed Working Memory on the WMS-III as a Marker for Poor Effort | Not known group design |
| 66 | Ross, 2006 | Psychological Disturbance, Incomplete Effort, and Compensation-Seeking Status as Predictors of Neuropsychological Test Performance in Head Injury | Not known group design |
| 67 | Root, 2006 | Detection of inadequate effort on the California Verbal Learning Test-Second edition: Forced choice recognition and critical item analysis | Not known group design |
| 68 | Richman, 2006 | Objective Tests of Symptom Exaggeration in Independent Medical Examinations | Not known group design |
| 69 | Sumanti, 2006 | Noncredible psychiatric and cognitive symptoms in a workers’ compensation “stress” claim sample | Not known group design |
| 70 | Sweet, 2006 |  | Not known group design |
| 71 | Vagnini, 2006 |  | Not known group design |
| 72 | Yanez, 2006 | Effects of severe depression on TOMM performance among disability-seeking outpatients | Not known group design |
| 73 | Yantz, 2006 | Potential for interpretation disparities of Halstead–Reitan neuropsychological battery performances in a litigating sample | Not known group design |
| 74 | Chafetz, 2007 | Malingering on the Social Security Disability Consultative Exam: A New Rating Scale | Not known group design |
| 75 | Egeland, 2007 | Differentiating Malingering from Genuine Cognitive Dysfunction Using the Trail Making Test-Ratio and Stroop Interference Scores | Not known group design |
| 76 | Gervais, 2007 | Development and Validation of a Response Bias Scale (RBS) for the MMPI-2 | Not known group design |
| 77 | Green, 2007 | The Pervasive Influence of Effort  on Neuropsychological Tests | Not known group design |
| 78 | Gill, 2007 | The Role of Effort Testing in Independent Medical Examinations | Not known group design |
| 79 | Howe, 2007 | Characterization of the Medical Symptom Validity Test in evaluation of clinically referred memory disorders clinic patients | Not known group design |
| 80 | Nelson, 2007 | Examination of the new MMPI-2 Response Bias Scale (Gervais): relationship with MMPI-2 validity scales | Not known group design |
| 81 | Sun, 2007 | Cost and Outcome Analyses on the Timing of First Independent Medical Evaluation in Patients with Work-Related Lumbosacral Sprain | Not known group design |
| 82 | Brasseaux, 2008 | The Relationship Between the Modified Somatic Perception Questionnaire and Dynamic Platform Posturography | Not known group design |
| 83 | Chafetz, 2008 | Malingering on the Social Security  Disability Consultative Exam: Predictors  and Base Rates | Not known group design |
| 84 | Gervais, 2008 | Differential sensitivity of the response bias scale (RBS) and MMPI-2 validity scales to memory complaints | Not known group design |
| 85 | Greve, 2008 | Classification accuracy of the Portland digit recognition test in persons claiming exposure to environmental and industrial toxins | Not known group design |
| 86 | Lax, 2008 | More than meets the eye, social, economic, and emotional impacts of work-related injury and illness | Not known group design |
| 87 | Smart, 2008 | Use of MMPI-2 to predict cognitive effort: A hierarchically optimal classification tree analysis | Not known group design |
| 88 | Schipper, 2008 |  | Not known group design |
| 89 | Caty, 2009 | Reproducibility of the ABILOCO Questionnaire and Comparison Between Self-Reported and Observed Locomotion Ability in Adult Patients with Stroke | Not known group design |
| 90 | Larrabee, 2009 |  | Not known group design |
| 91 | Merten, 2009 | Symptom Validity Testing in Claimants with Alleged Posttraumatic Stress Disorder: Comparing the Morel Emotional Numbing Test, the Structured Inventory of Malingered Symptomatology, and the Word Memory Test | Not known group design |
| 92 | Tsushima, 2009 | Comparison of MMPI-2 Validity Scales Among Compensation-Seeking Caucasian and Asian American Medical Patients | Not known group design |
| 93 | Thomas, 2009 | Let’s not get hysterical: comparing the MMPI-2 validity, clinical and RC scales in TBI litigants tested for effort | Not known group design |
| 94 | Greiffenstein, 2010 | MMPI-2 validity scores in defense- versus plaintiff-selected examinations: a repeated measures study of examiner effects | Not known group design |
| 95 | Schroeder, 2010 | Validation of the sentence repetition test as a measure of suspect effort | Not known group design |
| 96 | Tolin, 2010 | Detecting Symptom Exaggeration in Combat Veterans Using the MMPI–2 Symptom Validity Scales: A Mixed Group Validation | Not known group design |
| 97 | Wolfe, 2010 |  | Not known group design |
| 98 | Whiteside, 2010 | Relationship between suboptimal  cognitive effort and the clinical scales  of the Personality Assessment Inventory | Not known group design |
| 99 | Wygant, 2010 | Further Validation of the MMPI-2 and MMPI-2-RF Response Bias Scale: Findings from Disability and Criminal Forensic Settings | Not known group design |
| 100 | Chafetz, 2011 | The A-Test: A Symptom Validity Indicator Embedded Within a Mental Status Examination for Social Security  Disability | Not known group design |
| 101 | Chafetz, 2011 |  | Not known group design |
| 102 | Cooper, 2011 | Utility of the Mild Brain Injury Atypical Symptoms Scale as a Screening Measure for Symptom Over-Reporting in Operation Enduring Freedom/Operation Iraqi Freedom Service Members with Post-Concussive Complaints | Not known group design |
| 103 | Davis, 2011 | Cross-Validation of Picture Completion Effort Indices in Personal Injury Litigants and Disability Claimants | Not known group design |
| 104 | Green, 2011 | Comparison Between the Test of Memory Malingering (TOMM) and the Nonverbal Medical Symptom Validity Test (NV-MSVT) in Adults with Disability Claims | Not known group design |
| 105 | Green, 2011 | Comparison Between the Test of Memory Malingering  (TOMM) and the Nonverbal Medical Symptom Validity  Test (NV-MSVT) in Adults with Disability Claims | Not known group design |
| 106 | Hellings, 2011 | Homelessness and Response Styles on the Personality Assessment Inventory in a Compensation-Seeking Sample | Not known group design |
| 107 | Musso, 2011 | Development and Validation of the Stanford Binet-5 Rarely Missed Items- Nonverbal Index for the Detection of Malingered Mental Retardation | Not known group design |
| 108 | Tsushima, 2011 | Comparison of MMPI-2 Validity Scale Scores of Personal Injury Litigants and Disability Claimants | Not known group design |
| 109 | Whiteside, 2011 | Classification Accuracy of Multiple  Visual Spatial Measures in the Detection  of Suspect Effort | Not known group design |
| 110 | Wygant, 2011 | Association of the MMPI-2 Restructured Form (MMPI-2-RF) Validity Scales with Structured Malingering Criteria | Not known group design |
| 111 | Youngjohn, 2011 | Independent Validation of the MMPI-2-RF Somatic/ Cognitive and Validity Scales in TBI Litigants Tested for Effort | Not known group design |
| 112 | Armistead-Jehle, 2012 | Memory Complaints Inventory and Symptom Validity Test Performance in a Clinical Sample | Not known group design |
| 113 | Davis, 2012 | Performance Validity and Neuropsychological Outcomes in Litigants and Disability Claimants | Not known group design |
| 114 | Rogers, 2012 | The Detection of Feigned Disabilities: The Effectiveness of the Personality Assessment Inventory in a Traumatized Inpatient Sample | Not known group design |
| 115 | Whiteside, 2012 | Differential response patterns on the Personality Assessment Inventory (PAI) in compensation-seeking and non-compensation-seeking mild traumatic brain injury patients | Not known group design |
| 116 | Young, 2012 |  | Not known group design |
| 117 | Young, 2012 |  | Not known group design |
| 118 | Zakzanis, 2012 | The Predictive Utility of Neuropsychological Symptom Validity Testing as It Relates to Psychological  Presentation | Not known group design |
| 119 | Davis, 2013 | Number of impaired scores as a performance validity indicator | Not known group design |
| 120 | Johnson-Greene, 2013 |  | Not known group design |
| 121 | Larrosa, 2013 | A physiologic performance on dynamic posturography in work- related patients | Not known group design |
| 122 | Tarescavage, 2013 | Association Between the MMPI-2 Restructured Form (MMPI-2-RF) and Malingered Neurocognitive Dysfunction Among Non-Head Injury Disability Claimants | Not known group design |
| 123 | Van Dyke, 2013 | Assessing Effort: Differentiating Performance and Symptom Validity | Not known group design |
| 124 | Cottingham, 2014 | Apparent effect of type of compensation seeking (disability versus litigation) on performance validity scores may be due to other factors | Not known group design |
| 125 | Henry, 2014 (3134) |  | Not known group design |
| 126 | Larrabee, 2014 | False-Positive Rates Associated with the Use of Multiple Performance and Symptom Validity Tests | Not known group design |
| 127 | Love, 2014 | Specificity and False Positive Rates of the Test of Memory Malingering, Rey 15-Item Test, and Rey Word Recognition Test Among Forensic Inpatients with Intellectual Disabilities | Not known group design |
| 128 | Marcopulos, 2014 | Clinical decision making in response to performance validity test failure in a psychiatric setting | Not known group design |
| 129 | Bar-Hen, 2015 | Empirically derived algorithm for performance validity assessment embedded in a widely used neuropsychological battery: validation among TBI patients in litigation | Not known group design |
| 130 | Crighton, 2015 | Embedded Effort Scales in the Repeatable Battery for the Assessment of Neuropsychological Status: Do They Detect Neurocognitive Malingering? | Not known group design |
| 131 | Flaherty, 2015 | Limited usefulness of the Rey fifteen item test in detection of invalid performance in veterans suspected of mild traumatic brain injury | Not known group design |
| 132 | Fleming, 2015 | Welcoming a paradigm shift in occupational therapy: symptom validity measures and cognitive assessment | Not known group design |
| 133 | Lange, 2015 |  | Not known group design |
| 134 | Zottoli, 2015 |  | Not known group design |
| 135 | Copeland, 2016 | Relative Utility of Performance and Symptom Validity Tests | Not known group design |
| 136 | Grills, 2016 | Performance validity test and neuropsychological assessment battery screening module performances in an active-duty sample with a history of concussion | Not known group design |
| 137 | Armistead-Jehle, 2017 | Comparison of neuropsychological and balance performance validity testing | Not known group design |
| 138 | Belcher, 2017 | Functional and symptomatic assessment of medico-legal claims after upper limb injuries | Not known group design |
| 139 | Binder, 2017 | Determination of the smoking gun of intent: significance testing of forced choice results in social security claimants | Not known group design |
| 140 | Chmielewski, 2017 | The Comparative Capacity of the Minnesota Multiphasic Personality Inventory–2 (MMPI–2) and MMPI–2 Restructured Form (MMPI-2-RF) Validity Scales to Detect Suspected Malingering in a Disability Claimant Sample | Not known group design |
| 141 | Crighton, 2017 | The Generalizability of Overreporting Across Self-Report Measures: An Investigation With the Minnesota Multiphasic Personality Inventory–2–Restructured Form and the Personality Assessment Inventory in a Civil Disability Sample | Not known group design |
| 142 | Dretsch, 2017 | Evaluating the clinical utility of the Validity-10 for detecting amplified symptom reporting for patients with mild traumatic brain injury and comorbid psychological health conditions | Not known group design |
| 143 | Erdodi, 2017 | A single error is one too many: examining alternative cutoffs on trial 2 of the TOMM | Not known group design |
| 144 | Fazio, 2017 |  | Not known group design |
| 145 | Fuermaier, 2017 | Noncredible Cognitive Performance at Clinical Evaluation of Adult ADHD: An Embedded Validity Indicator in a Visuospatial Working Memory Test | Not known group design |
| 146 | Grossi, 2017 | Evaluation of the Response Bias Scale and Improbable Failure Scale in Assessing Feigned Cognitive Impairment | Not known group design |
| 147 | Gaasedelen, 2017 | Exploring the sensitivity of the Personality Assessment Inventory symptom validity tests in detecting response bias in a mixed neuropsychological outpatient sample | Not known group design |
| 148 | Hu, 2017 | The World Health Organization Disability Assessment Schedule 2.0 can predict the institutionalization of patients with stroke | Not known group design |
| 149 | Keesler, 2017 | Red flags in clinical interview may forecast invalid neuropsychological testing | Not known group design |
| 150 | Shura, 2017 | Symptom and Performance Validity with Veterans Assessed for Attention- Deficit/Hyperactivity Disorder (ADHD) | Not known group design |
| 151 | Boljanovic, 2018 | Psychosocial flag signs: impact on work status following a compensable shoulder injury |  |
| 152 | Bailey, 2018 | Clinical utility of the Rey 15-Item Test, recognition trial, and error scores for detecting noncredible neuropsychological performance in a mixed clinical sample of veterans | Not known group design |
| 153 | Huang, 2018 | World health organization disability assessment schedule 2.0 as an objective assessment tool for predicting return to work after a stroke | Not known group design |
| 154 | Fuermaier, 2018 | Is motor activity during cognitive assessment an indicator for feigned attention-deficit/hyperactivity disorder (ADHD) in adults? | Not known group design |
| 155 | Gervais, 2018 | Inconsistent Responding on the MMPI-2-RF and Uncooperative Attitude: Evidence from Cognitive Performance Validity Measures | Not known group design |
| 156 | Mossman, 2018 | Trial 1 Versus Trial 2 of the Test of Memory Malingering: Evaluating Accuracy Without a “Gold Standard” | Not known group design |
| 157 | Tylicki, 2018 | Comparability of Structured Interview of Reported Symptoms (SIRS) and Structured Interview of Reported Symptoms–Second Edition (SIRS-2) Classifications with External Response Bias Criteria | Not known group design |
| 158 | Webber, 2018 | **Further validation of the Test of Memory Malingering (TOMM) Trial 1 performance validity index: Examination of false positives and convergent validity** | Not known group design |
| 159 | Critchfield, 2019 | Cognitive impairment does not cause invalid performance: Analyzing performance patterns among cognitively unimpaired, impaired, and noncredible participants across six performance validity tests | Not known group design |
| 160 | Glassmire, 2019 | Examining False-Positive Rates of Wechsler Adult Intelligence Scale (WAIS-IV) Processing Speed-Based Embedded Validity Indicators Among Individuals with Schizophrenia Spectrum Disorders | Not known group design |
| 161 | Hoelzle, 2019 | Erroneous Conclusions: The Impact of Failing to Identify Invalid Symptom Presentation When Conducting Adult Attention-Deficit/Hyperactivity Disorder (ADHD) Research | Not known group design |
| 162 | Morey, 2019 | Examining a novel performance validity task for the detection of feigned attentional problems | Not known group design |
| 163 | Reyes, 2019 |  | Not known group design |
| 164 | Soble, 2019 | Evaluating the accuracy of the Wechsler Memory Scale-Fourth Edition (WMS-IV) logical memory embedded validity index for detecting invalid test performance | Not known group design |
| 165 | Stevens, 2019 | The Word Memory Test in medicolegal assessment: a measure of effort and malingering? | Not known group design |
| 166 | Kanser, 2020 | Detecting feigned traumatic brain injury with eye tracking during a test of performance validity | Not known group design |
| 167 | Menatti, 2020 | Limited Prediction of Performance Validity Using Embedded Validity Scales of the Neurobehavioral Symptom  Inventory in an mTBI Veteran Sample | Not known group design |
| 168 | Mikey, 2020 | Distress Tolerance and Symptom Severity as Mediators of Symptom Validity Failure in Veterans With PTSD | Not known group design |
| 169 | Nijdam-Jones, 2020 | Detection of feigned posttraumatic stress disorder: A meta-analysis of the Minnesota Multiphasic Personality Inventory-2 (MMPI-2) | Not known group design |
| 170 | Fuermaier, 2020 | Utility of an attention-based performance validity test for the detection of feigned cognitive dysfunction after acquired brain injury | Not known group design |
| 171 | Grossi, 2020 | Assessing Feigning With the Feigning Evaluation INtegrating Sources (FEINS) in a Forensic Psychiatric Sample | Not known group design |
| 172 | Gomez-Benito, 2020 | Disability in bipolar I disorder: Application of Mokken scaling analysis and the graded response model to the World Health Organization Disability Assessment Schedule 2.0 | Not known group design |
| 173 | Jansen, 2020 | Feigning memory impairment in a forced-choice task: Evidence from event-related potentials | Not known group design |
| 174 | Kraemer, 2020 | Minimizing Evaluation Time While Maintaining Accuracy: Cross-Validation of the Test of Memory Malingering (TOMM) Trial 1 and First 10-Item Errors as Briefer Performance Validity Tests | Not known group design |
| 175 | Merten, 2020 | Prevalence of overreporting on symptom validity tests in a large sample of psychosomatic rehabilitation inpatients | Not known group design |
| 176 | Nijdam-Jones, 2020 | Detection of feigned posttraumatic stress disorder: A meta-analysis of the Minnesota Multiphasic Personality Inventory-2 (MMPI-2) | Not known group design |
| 177 | Richey, 2020 | A Comparison of Performance Validity Measures in Predicting MMPI-2 Lie Scale Results | Not known group design |
| 178 | Tylicki, 2020 | Examination of the MMPI-3 over-reporting scales in a forensic disability sample | Not known group design |
| 179 | Wolf, 2020 | Psychometric Performance of the Miller Forensic Assessment of Symptoms Test (M-FAST) in Veteran PTSD Assessment | Not known group design |
| 180 | Kanser, 2021 | Utility of WAIS-IV Digit Span indices as measures of performance validity in moderate to severe traumatic brain injury | Not known group design |
| 181 | Lace, 2021 | Exploring the Structured Inventory of Malingered Symptomatology in Patients with Multiple Sclerosis | Not known group design |
| 182 | Olsen, 2021 | Cross-validation of the Invalid Forgetting Frequency Index (IFFI) from the Test of Memory Malingering | Not known group design |
| 183 | Ord, 2021 | Performance Validity and Symptom Validity Tests: Are They Measuring Different Constructs? | Not known group design |
| 184 | Rhoads, 2021 | Psychometric implications of failure on one performance validity test: a cross-validation study to inform criterion group definition | Not known group design |
| 185 | Schroeder, 2021 | Base rates of invalidity when patients undergoing routine clinical evaluations have social security disability as an external incentive | Not known group design |
| 186 | Smith, 2021 | The Utility of the Test of Memory Malingering Trial 1 in Differentiating Neurocognitive, Emotional, and Behavioral Functioning in a Pediatric  Concussion Population | Not known group design |
| 187 | Tewolde, 2021 | New cut-score to improve performance of the Memory Validity Profile (MVP) in heterogenous clinical populations | Not known group design |
| 188 | Tylicki, 2021 | A Comparison of the MMPI-2-RF and PAI Overreporting Indicators in a Civil Forensic Sample with Emphasis on the Response Bias Scale (RBS) and the Cognitive Bias Scale (CBS) | Not known group design |
|  | | | |
| 1 | Schmand, 1998 | Cognitive complaints in patients after whiplash injury: the impact of malingering | Studies not conducted in North America |
| 2 | Leark, 1999 | An investigation into the effects of malingering on the test of variables of attention (TOVA) in a college aged sample | Studies not conducted in North America |
| 3 | Langeluddecke, 2003 | Quantitative measures of memory malingering on the Wechsler Memory Scale—Third edition in mild head injury litigants | Studies not conducted in North America |
| 4 | Van Hout, 2003 | Suboptimal Performance on  Neuropsychological Tests in Patients with  Suspected Chronic Toxic Encephalopathy | Studies not conducted in North America |
| 5 | Langeluddecke, 2004 | Validation of the Rarely Missed Index (RMI) in Detecting Memory Malingering in Mild Head Injury Litigants | Studies not conducted in North America |
| 6 | Lange, 2005 | Ecological Validity of the WMS-III Rarely Missed Index in Personal Injury Litigation | Studies not conducted in North America |
| 7 | Van Hout, 2006 | Cognitive functioning in patients with suspected chronic toxic encephalopathy: evidence for neuropsychological disturbances after controlling for insufficient effort | Studies not conducted in North America |
| 8 | Singh, 2007 | [Malingering of psychiatric disorders: A review](https://www.researchgate.net/profile/Sandeep-Grover-2/publication/242076263_Malingering_of_Psychiatric_Disorders_A_Review/links/0c96052b92b0dd8739000000/Malingering-of-Psychiatric-Disorders-A-Review.pdf) | Studies not conducted in North America |
| 9 | Vilar-Lopez, 2007 | Detection of malingering in a Spanish population using three specific malingering tests | Studies not conducted in North America |
| 10 | Stevens, 2008 | Malingering and uncooperativeness in psychiatric and psychological assessment: Prevalence and effects in a German sample of claimants | Studies not conducted in North America |
| 11 | Vilar-Lopez, 2008 | Use of specific malingering measures in a Spanish sample | Studies not conducted in North America |
| 12 | Vilar-Lopez, 2008 | Malingering detection in a Spanish population with a known-groups design | Studies not conducted in North America |
| 13 | Blaskewitz, 2009 | Detection of Suboptimal Effort with the Rey Complex Figure Test and Recognition Trial | Studies not conducted in North America |
| 14 | Vetter, 2009 | The pattern of psychopathology associated with malingering tendencies at basic psychiatric screening of the Swiss Armed Forces | Studies not conducted in North America |
| 15 | Stevens, 2010 | Psychomotor Retardation: Authentic or Malingered? A Comparative Study of Subjects with and Without Traumatic Brain Injury and Experimental Simulators | Studies not conducted in North America |
| 16 | Shin, 2010 | Development of a Cognitive Level Explanation Model in Brain Injury : Comparisons between Disability and Non-Disability Evaluation Groups | Studies not conducted in North America |
| 17 | Soria, 2011 | Effectiveness of clinical assessment in Spanish forensic practice: detecting malingered psychological sequelae in victims of intimate partner violence | Studies not conducted in North America |
| 18 | Ortega, 2012 | A Bayesian Latent Group Analysis for Detecting Poor Effort in the Assessment of Malingering | Studies not conducted in North America |
| 19 | Webb, 2012 | Effort Test Failure: Toward a Predictive Model | Studies not conducted in North America |
| 20 | Belenguer-Prieto, 2013 | Specificity and sensitivity of objective tests to detect possible malingering in fibromyalgia: a case-control study in 211 Spanish patients | Studies not conducted in North America |
| 21 | Schindler, 2013 | Using the yes/no recognition response pattern to detect memory malingering | Studies not conducted in North America |
| 22 | Stevens, 2013 | Self-Report vs. Clinical Interview for Posttraumatic Stress Disorder in Medicolegal Assessment | Studies not conducted in North America |
| 23 | Sullivan, 2013 | A known-groups evaluation of the Response Bias Scale in a neuropsychological setting | Studies not conducted in North America |
| 24 | Zimmermann, 2013 | Detection of Malingering in the Assessment of Occupational Disability in the Military | Studies not conducted in North America |
| 25 | Hegedish, 2015 | Preliminary validation of a new measure of negative response bias: the temporal memory sequence test | Studies not conducted in North America |
| 26 | Keyvan, 2015 | The validity and reliability of the Turkish version of Miller Forensic Assessment of Symptoms Test (M-FAST) | Studies not conducted in North America |
| 27 | Stevens, 2016 | Reaction time as an indicator of insufficient effort: Development and validation of an embedded performance validity parameter | Studies not conducted in North America |
| 28 | Berufsverband der Augenarzte Deutschlands e, 2017 | Ophthalmological assessment in the Severely Handicapped Persons Act and in blindness: Statement of the Professional Association of Ophthalmologists Germany, the German Ophthalmological Society processed by the Joint Commission on Law | Studies not conducted in North America |
| 29 | Jung, 2017 | Association between working time quality and self-perceived health: Analysis of the 3rd Korean working conditions survey | Studies not conducted in North America |
| 30 | Kröger, 2017 | Evaluation of a visual acuity test using closed Landolt-Cs to determine malingering | Studies not conducted in North America |
| 31 | Meyer, 2017 | The visual association test-extended: a cross-sectional study of the performance validity measures | Studies not conducted in North America |
| 32 | Nijdam-Jones 2017 | A Cross-Cultural Analysis of the Test of Memory Malingering Among Latin American Spanish-Speaking Adults | Studies not conducted in North America |
| 33 | Plohmann, 2017 | Prevalence of poor effort and malingered neurocognitive dysfunction in litigating patients in Switzerland | Studies not conducted in North America |
| 34 | Torices, 2017 | Validation of neuropsychological consequences in victims of intimate partner violence in a spanish population using specific effort tests | Studies not conducted in North America |
| 35 | De Marchi, 2018 | Detecting malingering mental illness in forensics: Known-Group Comparison and Simulation Design with MMPI-2, SIMS and NIM | Studies not conducted in North America |
| 36 | Gomes, 2018 | Staying in the labor force among patients with rheumatoid arthritis and associated factors in Southern Brazil | Studies not conducted in North America |
| 37 | Mengel, 2018 | Costs of illness in chronic inflammatory demyelinating polyneuropathy in Germany | Studies not conducted in North America |
| 38 | Kuntz, 2018 | Social inequalities in the prevalence of chronic back pain among adults in Germany | Studies not conducted in North America |
| 39 | Ardic, 2019 | Reliability, validity, and factorial structure of the Turkish version of the Structured Inventory of Malingered Symptomatology (Turkish SIMS) | Studies not conducted in North America |
| 40 | Milon, 2019 | Scientific Uncertainty in Courts. A France-Germany Comparative Perspective on Litigation surrounding Hepatitis B Vaccination | Studies not conducted in North America |
| 41 | Pace, 2019 | Malingering Detection of Cognitive Impairment With the b Test Is Boosted Using Machine Learning | Studies not conducted in North America |
| 42 | Park, 2019 | Pre-injury job characteristics and return to work among injured workers in South Korea: differences by socio-demographic and injury-related characteristics | Studies not conducted in North America |
| 43 | Prümer, 2019 | Questioning the stereotype of the “malingering bureaucrat”: Absence from work in the public and private sector in Germany | Studies not conducted in North America |
| 44 | Roskes, 2019 | Malingering in the Psychiatric Emergency Department | Studies not conducted in North America |
| 45 | Schrader, 2019 | The Incidence of Diagnosis of Munchausen Syndrome, Other Factitious Disorders, and Malingering | Studies not conducted in North America |
| 46 | Shin, 2019 | Analysis of judicial precedents cases regarding skin cancer from 1997 to 2017 in Republic of Korea | Studies not conducted in North America |
| 47 | Weller, 2019 | Technology's impact on tasks of employees with disabilities in Germany | Studies not conducted in North America |
| 48 | Correa, 2020 | Malingering and defensiveness on the Spanish Personality Assessment Inventory: An initial investigation with mostly Spanish-speaking outpatients | Studies not conducted in North America |
| 49 | Geile, 2020 | Incidence of the diagnosis of factitious disorders–nationwide comparison study between Germany and Norway | Studies not conducted in North America |
| 50 | Giromini, 2020 | Using the inventory of problems - 29 (IOP-29) with the Test of Memory Malingering (TOMM) in symptom validity assessment: A study with a Portuguese sample of experimental feigners | Studies not conducted in North America |
| 51 | Giromini, 2020 | An Inventory of Problems–29 Sensitivity Study Investigating Feigning of Four Different Symptom Presentations Via Malingering Experimental Paradigm | Studies not conducted in North America |
| 52 | Gegner, 2021 | An Australian study on feigned mTBI using the Inventory of Problems – 29 (IOP-29), its Memory Module (IOP-M), and the Rey Fifteen Item Test (FIT) | Studies not conducted in North America |
| 53 | Grønnerød, 2023 | Validity of the Norwegian Version of Inventory of Problems–29 (IoP-29): A simulation study with experimental feigning of depression and a nonclinical control group. | Studies not conducted in North America |
| 54 | Merten, 2023 | [Laypeople's prevalence estimates of malingering: Survey data from the Netherlands.](https://psycnet.apa.org/getdoi.cfm?doi=10.1037/pne0000303) | Studies not conducted in North America |
| 55 | Puente-López, 2023 | [Prevalence estimates of symptom feigning and malingering in Spain](https://link.springer.com/article/10.1007/s12207-022-09458-w) | Studies not conducted in North America |
|  | | | |
| 1 | Slick, 1994 | Detecting dissimulation: Profiles of  simulated malingerers, traumatic brain-  injury patients, and normal controls on a  revised version of Hiscock and Hiscock’s  forced-choice memory test | Simulation study |
| 2 | Guilmette, 1996 | Order effects in the administration of a forced-choice procedure for detection of malingering in disability claimants’ evaluations’ | Simulation study |
| 3 | Griffin, 1996 | Assessing Dissimulation Among Social Security Disability Income Claimants | Simulation study |
| 4 | Tenhula, 1996 | Double Cross-Validation of the Booklet Category Test in Detecting Malingered Traumatic Brain Injury* | Simulation study |
| 5 | Chouinard, 1997 | The 48-Pictures Test: A two-alternative forced-choice recognition test for the detection of malingering | Simulation study |
| 6 | Suhr, 1999 | Use of the Wisconsin Card Sorting Test in the Detection of Malingering in Student Simulator and Patient Samples | Simulation study |
| 7 | Boone, 2005 | Comparison of various RAVLT scores in the detection of noncredible memory performance | Simulation study |
| 8 | Bianchini, 2008 | Classification Accuracy of MMPI-2 Validity Scales in the Detection of Pain-Related Malingering | Simulation study |
| 9 | King, 2009 | Deterring Malingered Psychopathology: The Effect of Warning Simulating Malingerers | Simulation study |
| 10 | Edmundson, 2017 | The Effects of Symptom Information Coaching on the Feigning of Adult ADHD | Simulation study |
| 11 | Smith, 2017 | Intentional Inattention: Detecting Feigned Attention-Deficit/Hyperactivity Disorder on the Personality Assessment Inventory | Simulation study |
| 12 | Viglione, 2017 | The Development of the Inventory of Problems–29: A Brief Self-Administered Measure for Discriminating Bona Fide From Feigned Psychiatric and Cognitive Complaints | Simulation study |
| 13 | Brand, 2019 | Detecting Clinical and Simulated Dissociative Identity Disorder with the Test of Memory Malingering | Simulation study |
| 14 | Clark, 2021 | Detecting simulated memory impairment in college students with the Progressive Visual Memory Test (PVMT): Validation of a new test of performance validity | Simulation study |
| 15 | Courrege, 2019 | The ADHD Symptom Infrequency Scale (ASIS): A Novel Measure Designed to Detect Adult ADHD Simulators | Simulation study |
| 16 | Berger, 2021 | Detection of Feigned ADHD Using the MOXO-d-CPT | Simulation study |
| 17 | Rogers, 2021 | Embedded WAIS-IV Detection Strategies and Feigned Cognitive Impairment: An Investigation of Malingered ADHD | Simulation study |
|  | | | |
| 1 | Lees-Haley, 1991 | MMPI-2 F and F-K scores of personal injury malingerers in vocational neuropsychological and emotional distress claims | Duplicate |
| 2 | Binder, 1993 | Assessment of malingering after mild  head trauma with the portland digit  recognition test | Duplicate |
| 3 | Greiffenstein, 1994 | Validation of Malingered Amnesia Measures with a Large Clinical Sample | Duplicate |
| 4 | Guilmette, 1996 | Order effects in the administration of a forced-choice procedure for detection of malingering in disability claimants’ evaluations’ | Duplicate |
| 5 | Larrabee, 1998 | Somatic Malingering on the MMPI and MMPI-2 in Personal Injury Litigants* | Duplicate |
| 6 | Meyers, 1998 | Validation of Reliable Digits for Detection of Malingering | Duplicate |
| 7 | Slick, 2000 | California Verbal Learning Test Indicators of Suboptimal Performance in a Sample of Head-Injury Litigants | Duplicate |
| 8 | Gervais, 2001 | Effects of coaching on symptom validity testing in chronic pain patients presenting for disability assessments | Duplicate |
| 9 | Green, 2001 | Effort has a greater effect on test scores than severe brain injury in compensation claimants | Duplicate |
| 10 | Greve, 2002 | Using the Wisconsin Card Sorting Test to Detect Malingering: An Analysis of the Specificity of Two Methods in Non malingering Normal and Patient Samples | Duplicate |
| 11 | Greve, 2003 | Detecting malingered performance on the Wechsler Adult Intelligence Scale Validation of Mittenberg's approach in traumatic brain injury | Duplicate |
| 12 | Gervais, 2004 | A comparison of WMT, CARB, and TOMM failure rates in non-head injury disability claimants | Duplicate |
| 13 | Etherton, 2006b | Pain, Malingering, and Performance on the WAIS-III Processing Speed Index | Duplicate |
| 14 | Greve, 2006a | Sensitivity and specificity of MMPI-2 validity scales and indicators to malingered neurocognitive dysfunction in traumatic brain injury | Duplicate |
| 15 | Greve, 2008a | Classification accuracy of the Portland digit recognition test in persons claiming exposure to environmental and industrial toxins | Duplicate |
| 16 | Green, 2011 | Comparison Between the Test of Memory Malingering (TOMM) and the Nonverbal Medical Symptom Validity Test (NV-MSVT) in Adults with Disability Claims | Duplicate |
| 17 | Bianchini, 2017 | The financial incentive effect: It's not just malingering | Duplicate |
| 18 | Borchman, 2017 | Examining the role of effort in embedded and self-report measures in the neuropsychological testing of traumatic brain injury | Duplicate |
| 19 | Hampton, 2017 | Psychopathy and malingering: Examining proneness to malinger in an inmate sample | Duplicate |
| 20 | Geba, 2017 | Assessment of malingered neurocognitive impairment: Examining the use of embedded measures in place of symptom validity tests | Duplicate |
| 21 | Ramachandran, 2017 | Identification of stimulant misuse and malingering of symptoms of attention deficit hyperactivity disorder | Duplicate |
| 22 | Nijdam-Jones 2017 | Cross-cultural feigning assessment: A systematic review of feigning instruments used with linguistically, ethnically, and culturally diverse samples | Duplicate |
| 23 | Nielsen, 2017 | Examining the relationship between depression and malingering in traumatic brain injury evaluations in a military population | Duplicate |
| 24 | Plohmann, 2017 | Prevalence of poor effort and malingered neurocognitive dysfunction in litigating patients in Switzerland | Duplicate |
| 25 | Viglione, 2017 | The Development of the Inventory of Problems–29: A Brief Self-Administered Measure for Discriminating Bona Fide From Feigned Psychiatric and Cognitive Complaints | Duplicate |
| 26 | Bianchini, 2018 | Classification accuracy of the Minnesota Multiphasic Personality Inventory-2 (MMPI-2)-Restructured form validity scales in detecting malingered pain-related disability | Duplicate |
| 27 | Granacher, 2018 | Feigned medical presentations | Duplicate |
| 28 | Garcia-Willingham, 2018 | Assessment of feigned cognitive impairment using standard neuropsychological tests | Duplicate |
| 29 | Frederick, 2018 | Feigned amnesia and memory problems | Duplicate |
| 30 | Rogers, 2018 | Clinical assessment of malingering and deception | Duplicate |
| 31 | Rogers, 2018 | Detection strategies for malingering and defensiveness | Duplicate |
| 32 | Merten, 2018 | False symptom claims and symptom validity assessment | Duplicate |
| 33 | Brand, 2019 | Detecting Clinical and Simulated Dissociative Identity Disorder With the Test of Memory Malingering | Duplicate |
| 34 | Critchfield, 2019 | Cognitive impairment does not cause invalid performance: Analyzing performance patterns among cognitively unimpaired, impaired, and noncredible participants across six performance validity tests | Duplicate |
| 35 | Ernst, 2019 | Detecting adult attention-deficit/hyperactivity disorder malingering using the behavior rating inventory of executive functioning-adult | Duplicate |
| 36 | Grossi, 2019 | Assessing feigning with the malingering assessment of psychopathology (map) in a forensic psychiatric sample | Duplicate |
| 37 | Golanics, 2019 | Malingering undetected successfully: Does extrinsic motivation and coaching have a significant impact? | Duplicate |
| 38 | McBride, 2019 | Latent class analysis of malingering classifications using performance and symptom validity measures in a civil forensic setting | Duplicate |
| 39 | Seybert-Williams, 2019 | The effectiveness of brief measures in screening for malingered chronic pain in a primary care setting | Duplicate |
| 40 | Wallace, 2019 | A meta-analysis of malingering detection measures for attention-deficit/hyperactivity disorder | Duplicate |
| 41 | Giromini, 2020 | Using the inventory of problems - 29 (IOP-29) with the Test of Memory Malingering (TOMM) in symptom validity assessment: A study with a Portuguese sample of experimental feigners | Duplicate |
| 42 | Kosky, 2020 | Methods of detection of feigned attention deficit hyperactivity disorder in a college-student population | Duplicate |
| 43 | Kanser, 2020 | Detecting feigned traumatic brain injury with eye tracking during a test of performance validity | Duplicate |
| 44 | Liberti, 2020 | Symptom Validity Testing | Duplicate |
| 45 | Lace, 2020 | Detecting feigned neurocognitive impairment related to mild traumatic brain injury: Comparing embedded effort indicators to standalone measures | Duplicate |
| 46 | Siegel, 2020 | The effects of incentivizing ADHD related non-credible responding on neuropsychological measures and performance validity tests | Duplicate |
| 47 | Regan, 2020 | Malingered cognitive symptoms in head injury | Duplicate |
| 48 | Nijdam-Jones, 2020 | Detection of feigned posttraumatic stress disorder: A meta-analysis of the Minnesota Multiphasic Personality Inventory-2 (MMPI-2) | Duplicate |
| 49 | Clark, 2021 | Detecting simulated memory impairment in college students with the Progressive Visual Memory Test (PVMT): Validation of a new test of performance validity | Duplicate |
