# Supplementary material for: Prevalence of symptom exaggeration among North American independent medical evaluation examinees: A systematic review of observational studies
Source: PLoS One. 2025 Jun 25;20(6):e0324684. doi: 10.1371/journal.pone.0324684 (PMC12193048; doi:10.1371/journal.pone.0324684)
Supplement: S6 Table — (DOCX) [file pone.0324684.s006.docx]

**S6 Table:** Data extracted from included studies

| **Author** | **Reviewers** | **Date of extraction** | **Diagnosis** | **Age (Mean)** | **Age (SD)** | **% female** | **Education (years)** | **Mild severity (%)** | **Denominator** | **Events** |  |
| --- | --- | --- | --- | --- | --- | --- | --- | --- | --- | --- | --- |
|  |  |  |  |  |  |  |  |  |  |  |  |
| Lees-Haley, 1991 | AD, AL | 21/8/2023 | personal-injury claimants | 37.78 | 11.35 | 57.78 | NR | NA | 45 | 25 |  |
| Greiffenstein, 1995 | AD, AL | 18/8/2023 | TBI | 35.43 | 11.44 | NR | 12.13 | NR | 121 | 68 |  |
| Suhr, 1997 | AD, AL | 22/8/2023 | TBI | 35.3 | 9.11 | 45.75 | 13.17 | 63.5 | 96 | 31 |  |
| Costa, 1999 | AD, RM | 25/8/2023 | memory impairment | 40.74 | 8.84 | 54.76 | 11.52 | NR | 42 | 28 |  |
| van Gorp, 1999 | AD, RM | 27/8/2023 | TBI | 36.62 | 12.74 | NR | 13.33 | NR | 81 | 20 |  |
| Sweet, 2000 | AD, AL | 22/8/2023 | TBI | 37.43 | 11.71 | NR | 13.8 | NR | 63 | 21 |  |
| Greve, 2003 | AD, AL | 16/8/2023 | TBI | 36.61 | 13.74 | 33.85 | 12.83 | NR | 113 | 28 |  |
| Lu, 2003 | AD, RM | 25/8/2023 | TBI and mixed neurological (other than TBI) | 42.51 | 12.60 | 43.75 | 12.85 | NR | 128 | 58 |  |
| Barrash, 2004 | AD, RM | 20/8/2023 | TBI and mixed neurological (other than TBI) | 45.19 | 14.20 | 53.70 | 12.88 | NR | 108 | 25 |  |
| Heinly, 2005 | AD, AL | 21/8/2023 | TBI | 39.60 | 13.80 | 30.00 | 12.10 | 57 | 344 | 71 |  |
| Curtis, 2006 | AD, AL | 16/8/2023 | TBI | 38.71 | 12.91 | 28.00 | 12.27 | 26 | 275 | 55 |  |
| Etherton, 2006a | AD, AL | 16/8/2023 | Chronic pain | 43.3 | 10.61 | 35.8 | 11.9 | NA | 81 | 32 |  |
| Greve, 2006a | AD, AL | 18/8/2023 | TBI | 38.70 | 12.40 | 29 | 12.50 | 61.8 | 259 | 45 |  |
| Greve, 2006b | AD, AL | 18/8/2023 | TBI | 39.3 | 12.9 | 27 | 12.3 | 63.98 | 161 | 41 |  |
| Greve, 2006c | AD, AL | 18/8/2023 | TBI | 38.3 | 12.7 | 27.00 | 12.2 | 58.02 | 262 | 56 |  |
| Greve, 2006d | AD, AL | 18/8/2023 | Cognitive dysfunction upon exposure to occupational and environmental substances | 40.80 | 11.10 | 28.1 | 12.00 | NA | 120 | 48 |  |
| Ardolf, 2007 | AD, RM | 25/8/2023 | TBI and mixed neurological (other than TBI) | 40.07 | 13.07 | 0.00 | 10.54 | NR | 105 | 57 |  |
| Greve, 2007 | AD, RM | 22/8/2023 | TBI | 39 | 12.1 | 30 | 12.6 | 63.11 | 206 | 59 |  |
| Henry, 2007 | AD, RM | 18/8/2023 | TBI and mixed neurological (other than TBI) | 39.80 | 12.63 | 46.30 | 14.32 | NR | 54 | 27 |  |
| O'Bryant, 2007 | AD, AL | 21/8/2023 | TBI and mixed neurological (other than TBI) | 41.00 | 13.20 | 32.52 | 12.70 | NR | 329 | 98 |  |
| Greve, 2007a | AD, AL | 18/8/2023 | Toxic exposure | 41.28 | 10.84 | 28.46 | 12.04 | NA | 123 | 46 |  |
| Aguerrevere, 2008 | AD, RM | 20/8/2023 | TBI | 37.83 | 1.74 | 28.42 | 12.42 | NR | 185 | 94 |  |
| Curtis, 2008 | AD, RM | 22/8/2023 | TBI | 39.6 | 13.8 | 28.9 | 12.3 | 52.94 | 204 | 65 |  |
| Greve, 2008 | AD, RM | 20/8/2023 | TBI | 38.30 | 13.60 | 28.40 | 12.10 | 60.19 | 211 | 56 |  |
| Ord, 2008 | AD, AL | 21/8/2023 | TBI | 36.19 | 11.23 | 35.50 | 12.67 | 69.9 | 93 | 31 |  |
| Greve, 2008b | AD, AL | 20/8/2023 | TBI | 40.35 | 11.16 | 24 | 12.27 | 67 | 109 | 27 |  |
| Greve, 2008b | AD, AL | 20/8/2023 | Chronic pain | 42.45 | 9.71 | 34.68 | 11.84 | NA | 228 | 58 |  |
| Henry, 2009 | AD, RM | 18/8/2023 | TBI and mixed neurological (other than TBI) | 42.03 | 14.20 | 40.99 | 13.83 | NR | 161 | 84 |  |
| Greve, 2009 | AD, RM | 20/8/2023 | TBI | 37.7 | 14.67 | 27.15 | 11.7 | 51.77 | 282 | 50 |  |
| Greve, 2009a | AD, AL | 20/8/2023 | Chronic pain | 41.2 | 8.8 | 35 | 11.8 | NA | 318 | 109 |  |
| Greve, 2009b | AD, AL | 18/8/2023 | TBI | 38.7 | 12.4 | 28.5 | 12.3 | 63.3 | 442 | 100 |  |
| Greve, 2009b | AD, AL | 18/8/2023 | Chronic pain | 42.4 | 9.5 | 36.8 | 11.6 | NA | 378 | 94 |  |
| Greve, 2009c | AD, AL | 20/8/2023 | Chronic pain | 42.3 | 9 | 36.3 | 11.7 | NA | 604 | 216 |  |
| Greve, 2009d | AD, AL | 20/8/2023 | Chronic pain | 42.10 | 9.60 | 35.40 | 11.60 | NA | 508 | 128 |  |
| Bortnik, 2010 | AD, RM | 22/8/2023 | TBI and mixed neurological (other than TBI) | 42.66 | 13.09 | 48.93 | 11.99 | NR | 188 | 63 |  |
| Curtis, 2010 | AD, RM | 25/8/2023 | TBI and mixed neurological (other than TBI) | 36.3 | 9.6 | 35.1 | 13 | 68.9 | 74 | 27 |  |
| Greve, 2010 | AD, AL | 18/8/2023 | Chronic pain | 41.10 | 8.90 | 35.00 | 11.70 | NA | 612 | 185 |  |
| Ord, 2010 | AD, AL | 22/8/2023 | TBI | 39.41 | 11.86 | 36.67 | 13.02 | 71.4 | 84 | 27 |  |
| Aguerrevere, 2011 | AD, AL | 16/8/2023 | TBI | 39.90 | 11.37 | 26.20 | 12.41 | 71 | 108 | 56 |  |
| Roberson, 2013 | AD, RM | 27/8/2023 | TBI and mixed neurological (other than TBI) | 43.08 | 12.11 | 44.44 | 13.13 | 23.17 | 315 | 212 |  |
| Bianchini, 2014 | AD, RM | 27/8/2023 | Chronic pain | 43.30 | 12.00 | 34.90 | 12.10 | NA | 328 | 105 |  |
| Guise, 2014 | AD, RM | 20/8/2023 | TBI | 38.25 | 12.45 | 31.00 | 12.61 | 61 | 126 | 41 |  |
| Patrick,2014 | AD, RM | 27/8/2023 | TBI and mixed neurological (other than TBI) | 43.50 | 11.29 | 17.31 | 13.13 | NR | 52 | 23 |  |
| Aguerrevere, 2017 | AD, RM | 27/8/2023 | Chronic pain | 43.10 | 8.70 | 35.63 | 11.70 | NA | 348 | 180 |  |
| Bianchini, 2018 | AD, RM | 27/8/2023 | Chronic pain | 42.27 | 8.92 | NR | 11.31 | NA | 501 | 214 |  |
| Curtis, 2019 | AD, AL | 16/8/2023 | Chronic pain | 43.49 | 9.73 | 32.40 | 12.22 | NA | 219 | 78 |  |
