# Supplementary figures and images for: Prevalence of symptom exaggeration among North American independent medical evaluation examinees: A systematic review of observational studies
Source: PLoS One. 2025 Jun 25;20(6):e0324684. doi: 10.1371/journal.pone.0324684 (PMC12193048; doi:10.1371/journal.pone.0324684)

**S1 Figure:** Meta-regression for proportion of females among 42 studies (p=0.16)


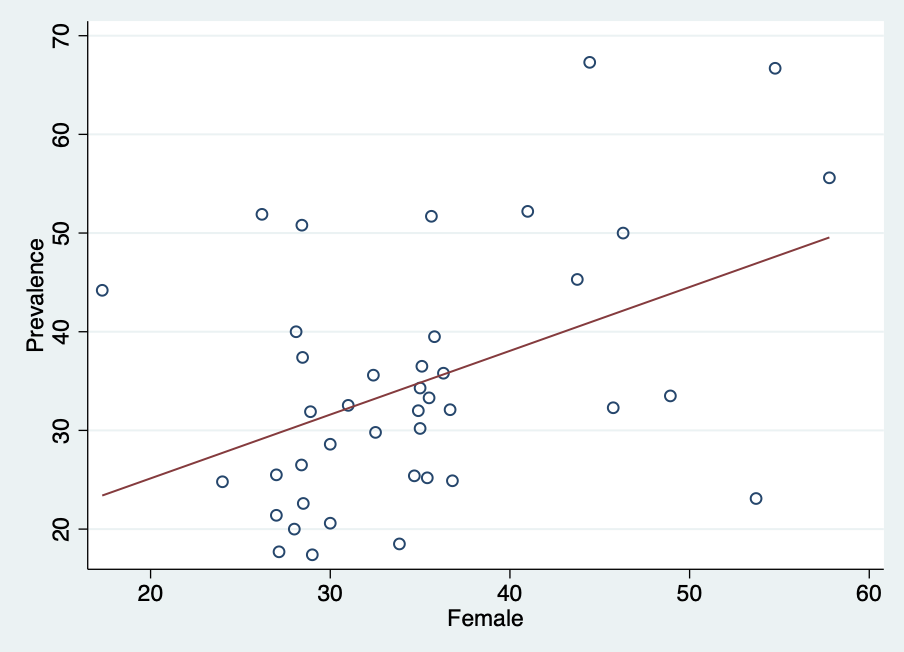


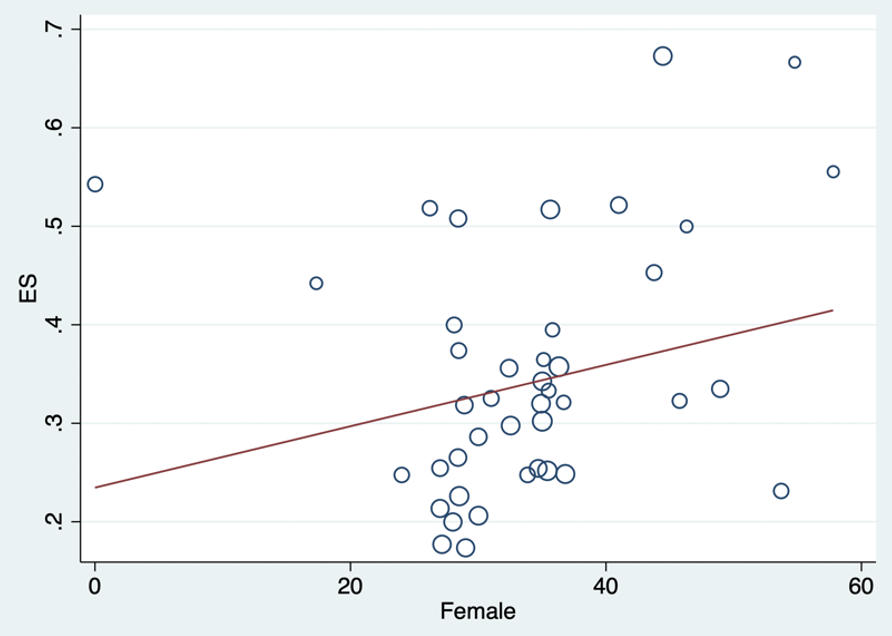


(95% CI -0.001 to 0.008; p = 0.160; tau2 = 0.009; I-squared_res = 65.32%)

Supplement: S1 Fig — (DOCX) [file pone.0324684.s007.docx]

**S3 Figure:** Subgroup analysis for type of conditions (test of interaction p=0.95)

**
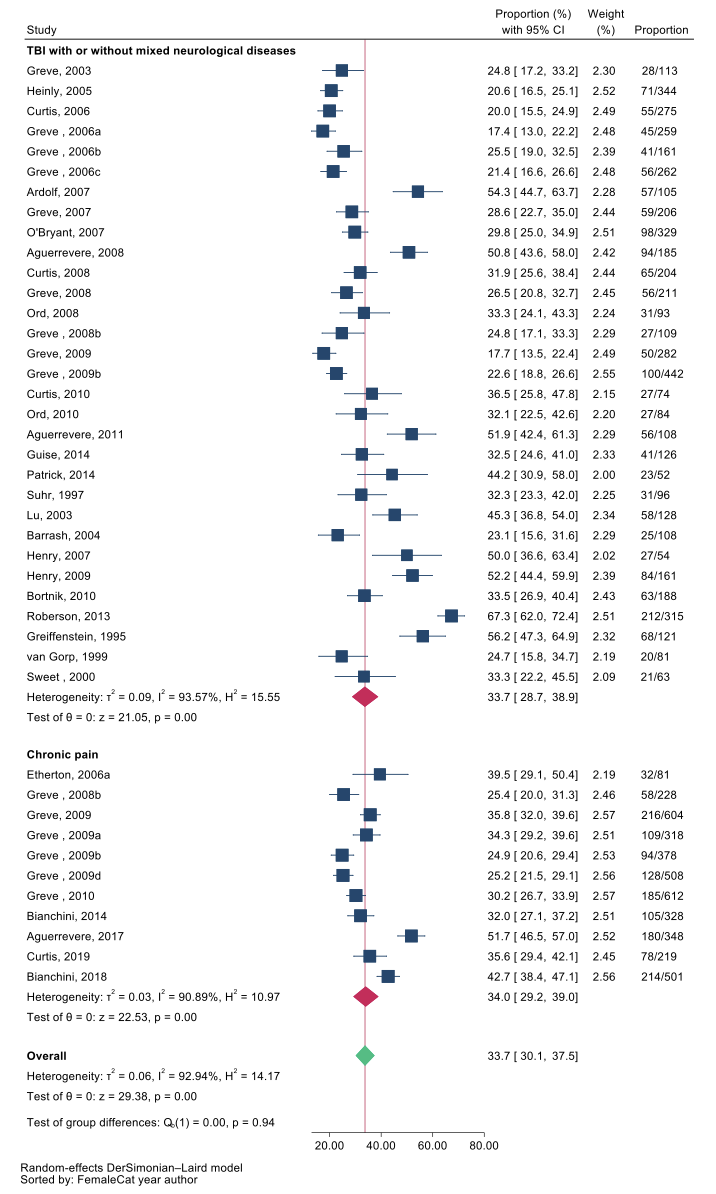
**

Supplement: S3 Fig — (DOCX) [file pone.0324684.s009.docx]

**S4 Figure:** Subgroup analysis for confidence in reference standard (test of interaction p=0.84)

**
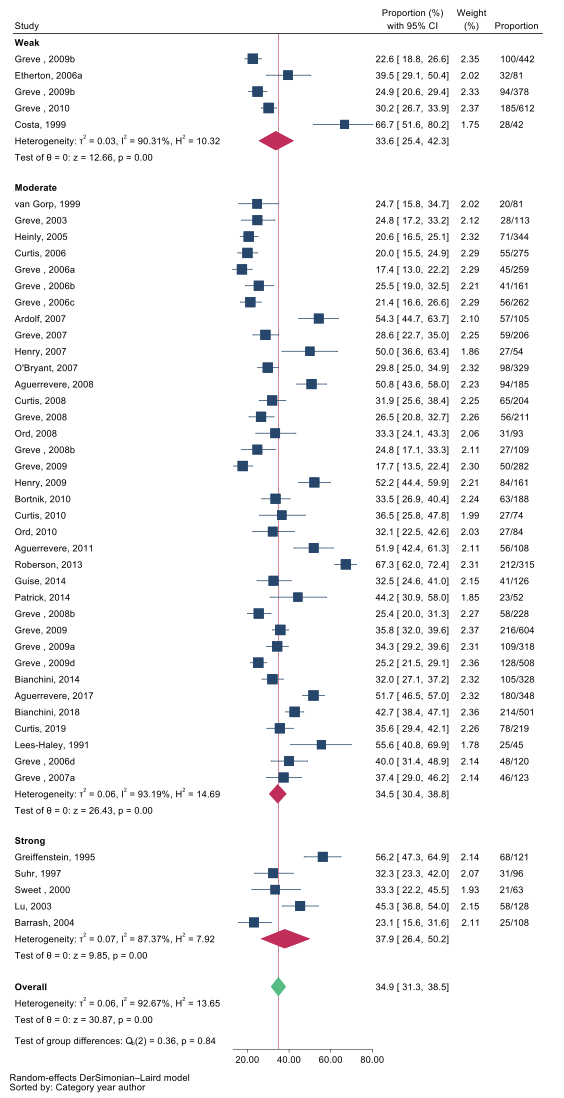
**

Supplement: S4 Fig — (DOCX) [file pone.0324684.s010.docx]

**S6 Figure:** Meta-regression for average age among 46 cohorts (p=0.18)


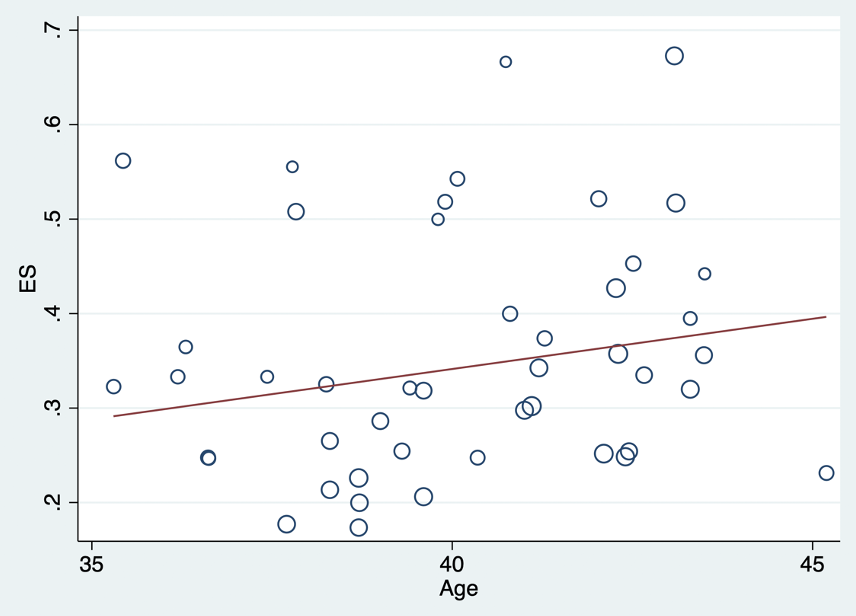


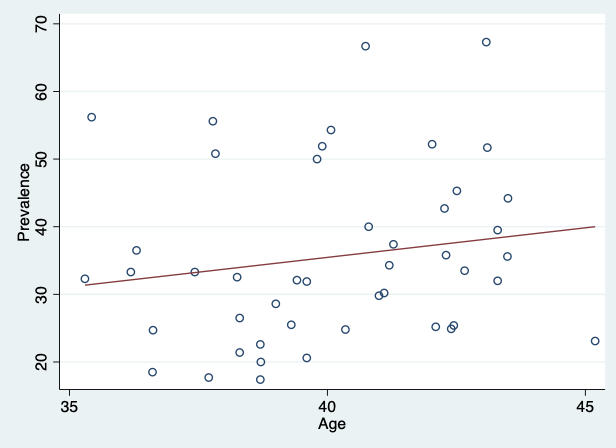


(95% CI -0.005- 0.026; p= 0.18; tau2= 0.009; I-squared res= 65.10%)

Supplement: S6 Fig — (DOCX) [file pone.0324684.s012.docx]

**S7 Figure:** Meta-regression for average education level among 45 cohorts (p=0.65)

**
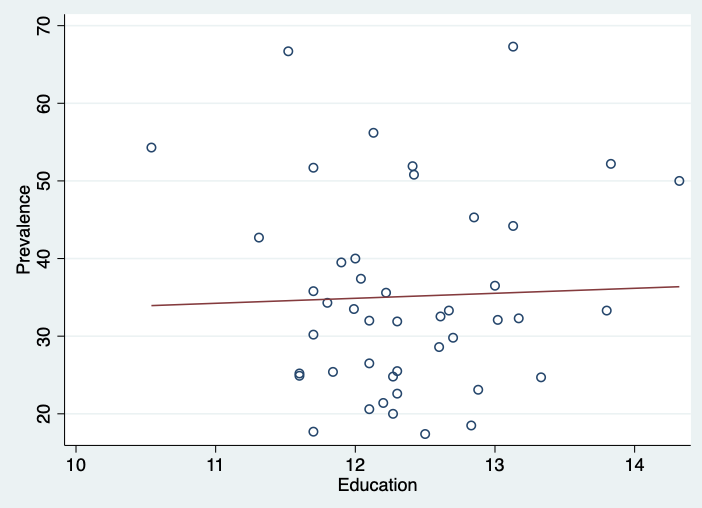
**

**
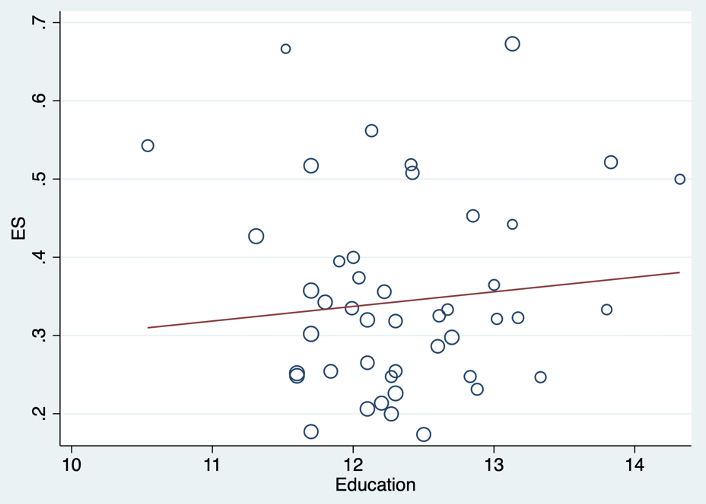
**

(95% CI -0.039- 0.077; p= 0.65; tau2= 0.009; I-squared_res= 67.81%)

Supplement: S7 Fig — (DOCX) [file pone.0324684.s013.docx]

**S2 Checklist:** Meta-analysis of Observational Studies in Epidemiology (MOOSE) checklist

**
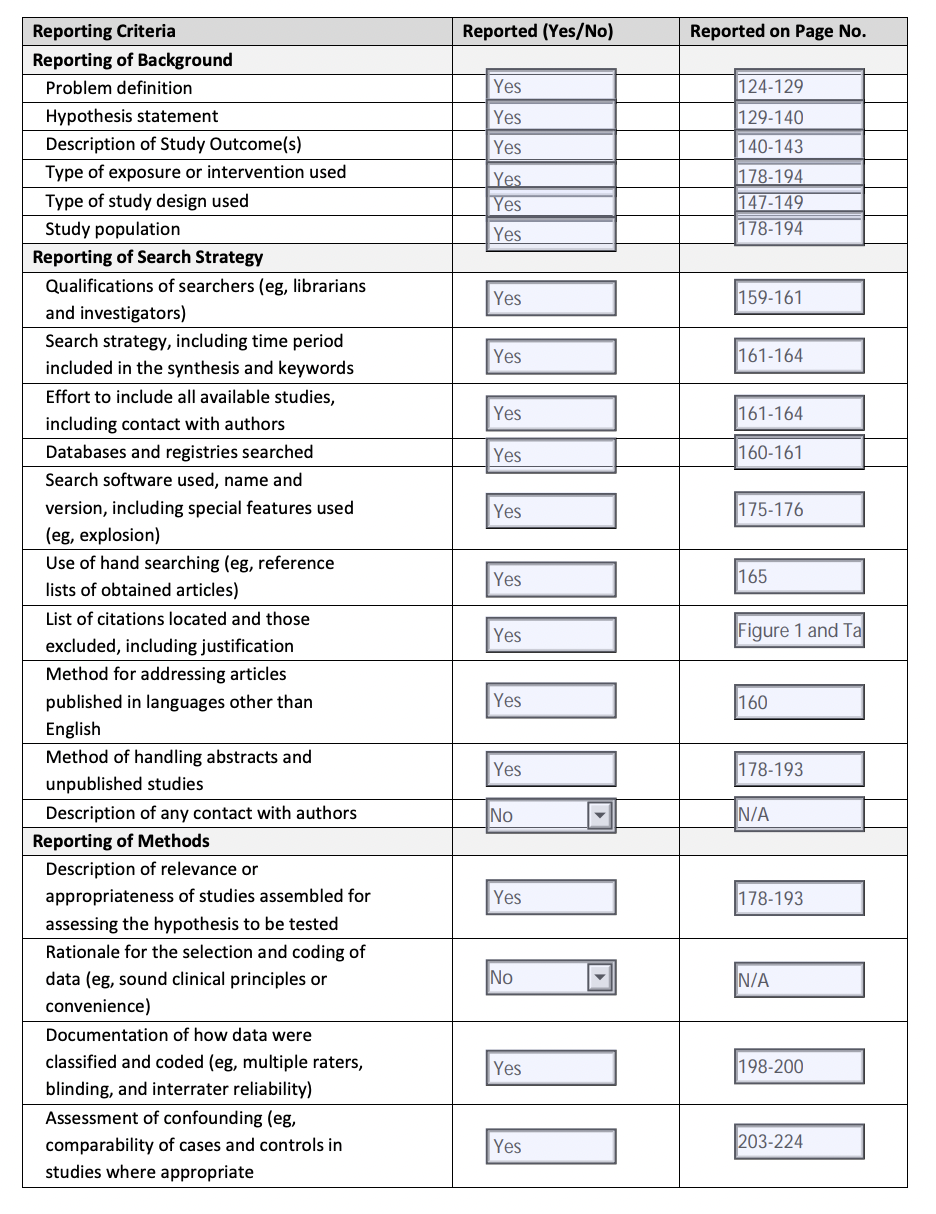
**

**
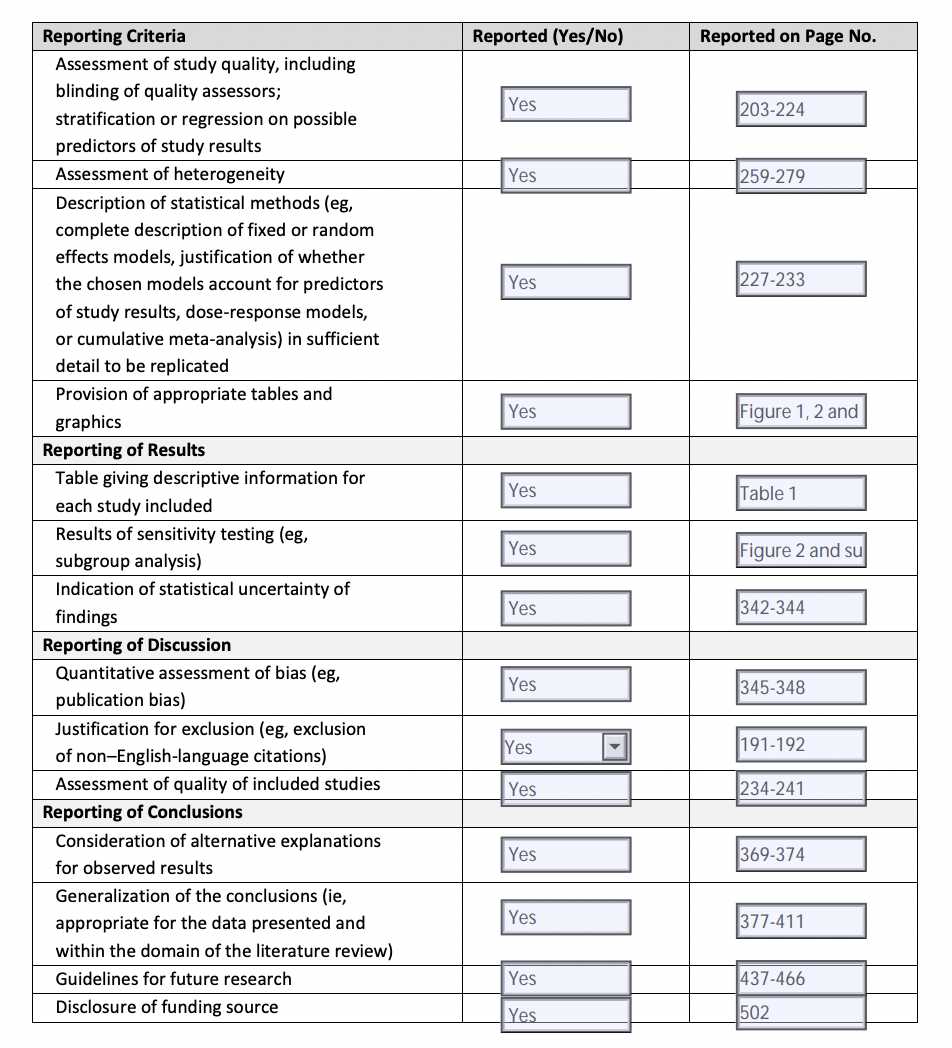
**

Supplement: S2 Checklist — (DOCX) [file pone.0324684.s015.docx]
