# Supplementary material for: Prevalence of symptom exaggeration among North American independent medical evaluation examinees: A systematic review of observational studies
Source: PLoS One. 2025 Jun 25;20(6):e0324684. doi: 10.1371/journal.pone.0324684 (PMC12193048; doi:10.1371/journal.pone.0324684)
Supplement: S2 Fig — (DOCX) [file pone.0324684.s008.docx]

**S2 Figure: a-** Funnel plots of overall prevalence (Egger’s test p= 0.13) and b- prevalence in subgroup of studies with female proportion <40% (Egger’s test p= 0.16)

**S2 Figure a (n=46 studies)**

**
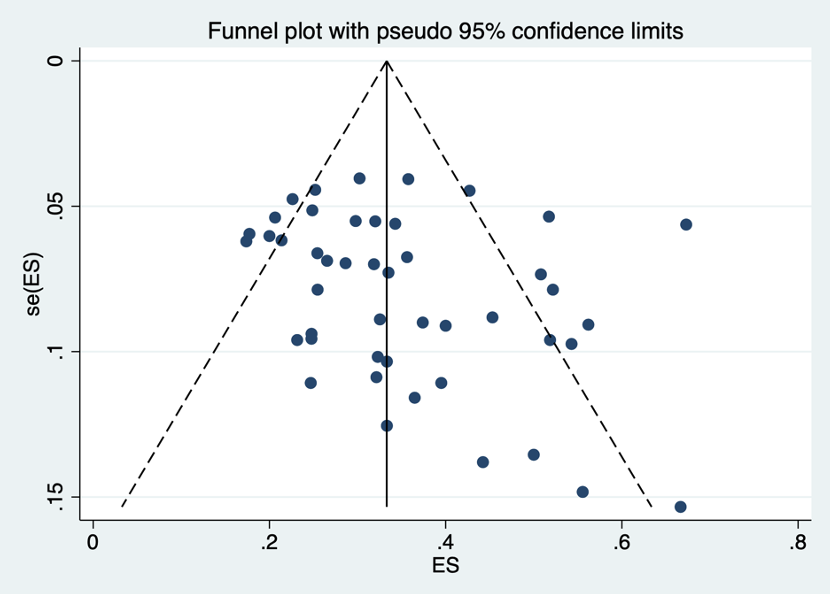
**

**S2 Figure b (n=33 studies)**

**
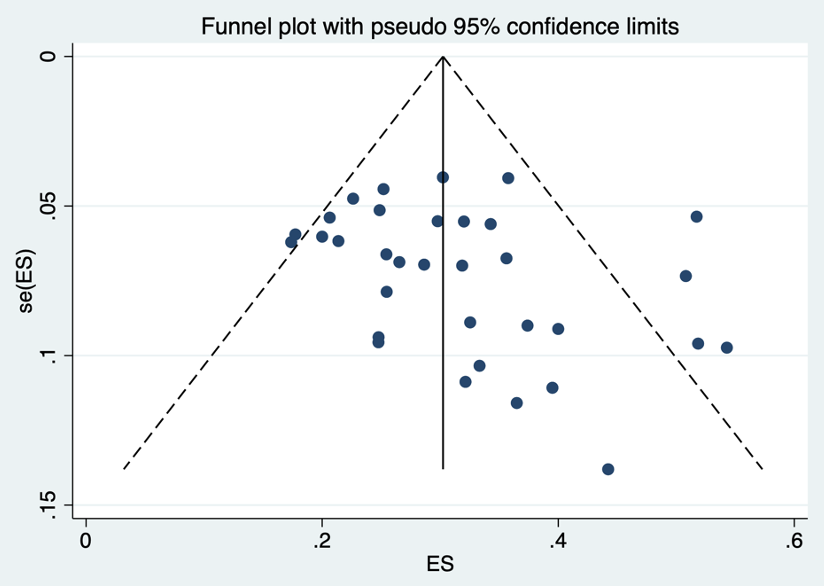
**
