# Supplementary material for: Prevalence of symptom exaggeration among North American independent medical evaluation examinees: A systematic review of observational studies
Source: PLoS One. 2025 Jun 25;20(6):e0324684. doi: 10.1371/journal.pone.0324684 (PMC12193048; doi:10.1371/journal.pone.0324684)
Supplement: S5 Fig — (DOCX) [file pone.0324684.s011.docx]

**S5 Figure:** Subgroup analysis for similar age and/or education between groups (test of interaction p=0.47)

**
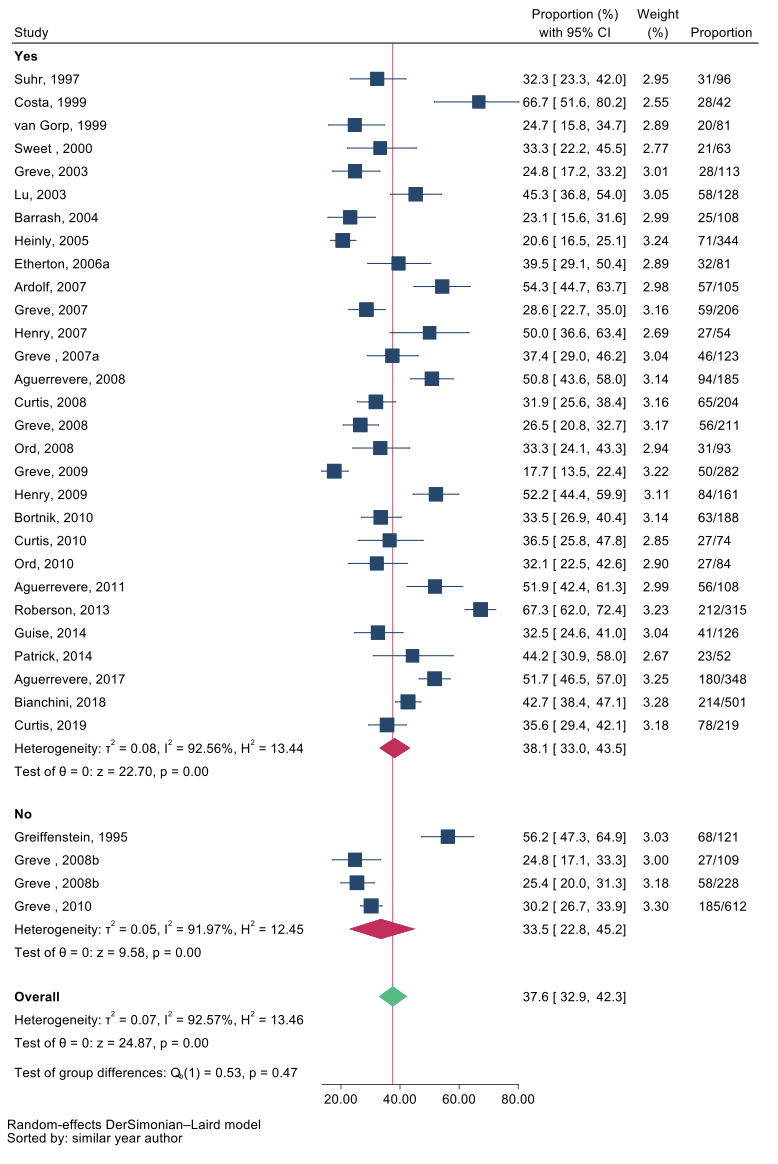
**
