# Supplementary material for: Prevalence of symptom exaggeration among North American independent medical evaluation examinees: A systematic review of observational studies
Source: PLoS One. 2025 Jun 25;20(6):e0324684. doi: 10.1371/journal.pone.0324684 (PMC12193048; doi:10.1371/journal.pone.0324684)
Supplement: Attachment — Submitted filename: IME_ Response to Reviewers_(Nov 21_2024).docx [file pone.0324684.s016.docx]

**Academic Editor**

Thank you for submitting your manuscript to PLOS ONE. After careful consideration, we feel that it has merit but does not fully meet PLOS ONE’s publication criteria as it currently stands. Therefore, we invite you to submit a revised version of the manuscript that addresses the points raised during the review process.

**Reply: Thank you for your feedback, and that of the reviewers. We have considered all recommendations and comments and have addressed them in our line-by-line responses below.**

**General Comments:**

1. Thank you for your submission. I read it with enthusiasm, as it shows notable merits. Nevertheless, I noticed some concerns that I'd like the authors to address.

**Reply 1: Thank you for your review and comments. We have addressed your concerns line-by-line below.**

2. Please double-check grammar (e.g. punctuation and verb tense)

**Reply 2: Thank you for your comment. We have reviewed the manuscript for grammatical errors.**

3. Please double-check refs (e.g. keep the requested style, Vancouver, and correct formatting, punctuation and remove repetitives)

**Reply 3: Thank you for this observation. We made the appropriate revisions to our references using tracked changes in the attached manuscript.**

**Abstract related comments**

4. The Abstract is well-structured but could benefit from conciseness. The inclusion of multiple phrases, such as "symptom exaggeration" and "observational studies," makes it somewhat repetitive. I'd suggest reducing redundant terms to improve readability.
**Reply 4: Thank you for your comment. We have taken your suggestion and made our abstract more concise including the removal of redundant terms and phrases.**

5. The logical transitions between the background, methods, and results are somewhat abrupt. For instance, it's unclear how the analysis of "symptom exaggeration" directly links to "inter-rater reliability" or whether the gender-specific findings are derived from a subset analysis or the primary outcomes. Please provide a clearer link between the methodological approach and the primary research question

**Reply 5: We appreciate your feedback. We undertook an assessment of the prevalence of symptom exaggeration, as if it was common it may help explain the poor inter-rater reliability seen in IME assessments. By quantifying the prevalence of symptom exaggeration, clinicians and policymakers can better understand the scope of this issue and work toward improving the objectivity and reliability of IMEs. We added the following to the abstract under the introduction section:**

**“*This study explored the prevalence of symptom exaggeration among IME examinees in North America, which if common would represent an important consideration for improving the reliability of IMEs.”***

**As for clarifying the methods related to the gender-specific findings we added the following in the abstract under the methods section:**

**“*We …. explored potential subgroup effects for sex, age, education, clinical condition, and confidence in the reference standard*.”**

6. The stats parameters (e.g. p-values, effect sizes, CIs) are provided, but more detail on the significance of these values will enhance the depth of the findings. For example, how do these rates of symptom exaggeration in IMEs impact policy, clinical practice, or future research directions? Offering these insights would broaden the reliability.

**Reply 6: Thank you. The low certainty in the findings highlighted the need for further research which we added to the conclusion of the abstract which now reads as follows:**

**“*The high prevalence of symptom exaggeration among IME attendees provides a compelling rationale for IME evaluators to formally explore this issue. Future research should establish the reliability and validity of current evaluation criteria for symptom exaggeration and develop a structured IME assessment approach*.”**

7. Sample size details are provided, but the inclusion of additional information, such as variations across cohorts or specific diagnostic categories, would be useful. For instance, were patients with traumatic brain injuries or chronic pain assessed differently? This would offer a clearer understanding of subgroup analysis and generalisation.

**Reply 7: Thank you. We have added clarifications, while attempting to remain concise, under the methods and results sections of the abstract. In terms of methods the added text is as noted in our response to comment 5. As for additions to the results, it now reads as follows:**

**“*We found no significant subgroup effects for type of clinical condition, confidence in the reference standard, age, or education*.”**

**8.** A brief explanation of whether the studies had high or low certainty is important.

# Reply 8: Thank you for your comment. We made clarifications to the results section by (1) describing the certainty of the body of evidence for our outcome of interest and (2) using informative statements to communicate our findings as per GRADE guidelines 26 (<https://www.sciencedirect.com/science/article/pii/S0895435619304160>). The language was revised in the abstract under the results section as follows:

**“*We found low certainty evidence suggesting that studies with a greater proportion of women (≥40% vs. <40%) may be associated with higher rates of exaggeration: 47% (95%CI 36 to 58) vs. 31% (95%CI 28 to 35; test of interaction p=0.02).*”**

**9.** The abstract briefly touches on gender differences but would benefit from elaborating on why women show higher rates of symptom exaggeration in these studies. Consider a brief sentence.

**Reply 9: Thank you for this observation. We added a sentence to the Results section of the abstract as follows:**

**“*This difference may be due to biological differences, greater bodily awareness, or higher rates of negative affectivity*.”**

10. The findings could be expanded to include more specific quantitative details, such as the range of exaggeration percentages across the cohorts or any significant moderators or predictors found in the meta-analysis.

**Reply 10: We agree. We have revised and added more findings to our results section of the abstract as follows:**

**“*Prevalence of symptom exaggeration across studies ranged from 17% to 67%. We found low certainty evidence suggesting that studies with a greater proportion of women (≥40% vs. <40%) may be associated with higher rates of exaggeration: 47% (95%CI 36 to 58) vs. 31% (95%CI 28 to 35; test of interaction p=0.02). We found no significant subgroup effects for type of clinical condition, confidence in the reference standard, age, or education*.”**

11. The conclusion is concise but could be expanded to include a brief part on how symptom exaggeration influences clinical decision-making during IMEs. Additionally, mentioning how these findings could influence future research or policy changes is essential;
**Reply 11: As noted in our response to comment 6 above, we added information to the conclusion section to expand on the implications of the findings on research and practice.**

12. The abstract would be improved by providing the main strengths, such as prevalence rates for symptom exaggeration or differences in exaggeration across different conditions;
**Reply 12: We appreciate this feedback and have added information on the range of prevalence rates across studies and noted that we did not find a credible subgroup effect between the overall prevalence and different clinical conditions, confidence in the reference standard, age, or education. This was added to the results section of the abstract as described above in response to comment number 10.**

13. The term "symptom exaggeration" is mentioned without any clear definition. Please elaborate

**Reply 13: We added a short statement to the background of the abstract as follows:**

**“*…This may be affected by symptom exaggeration where patients may feel pressure to fully convey their level of impairment to qualify for incentives*.”**

**Introduction and objective related comments**

14. The transition between the background on disabilities and the introduction of IMEs feels somewhat confusing. Providing a smoother transition into how symptom exaggeration affects IMEs will improve your argument;

- There are relevant stats about disabilities in North America, which effectively establishes the context. However, there is a need for a more explicit link between this background information and the objective. For example, while the IME process is mentioned, the transition to the issue of symptom exaggeration is quite abrupt

**Reply 14:** **We have considered your feedback and added text (that is underlined below) to further clarify our objective. The last paragraph of our introduction now reads as follows:**

**“*Patients referred for IMEs often present with subjective complaints (e.g., mental illness, chronic pain) and may feel pressure to emphasize their level of impairment to qualify for wage replacement benefits, receiving time off work, or other incentives (3, 9, 10). Whether or not IME assessors consider symptom exaggeration therefore has the potential to lead to very different conclusions; however, the prevalence of exaggeration among IME attendees is uncertain and individual studies report rates as low as 18% or as high as 68%. To address this gap in the literature, we undertook the first systematic review of observational studies to explore the prevalence of symptom exaggeration among IME examinees in North America.*”**

15. The Introduction touches on symptom exaggeration as a factor affecting IME outcomes. Nevertheless, there is the need to state how the authors would measure it or what variables will be examined (e.g. mental health, pain severity).

**Reply 15: We agree and described in our methods section the types of studies and criteria used that we considered eligible in this study as noted below:**

**“*As there is no singular reliable and valid criteria (reference standard) in the literature that is used to assess for symptom exaggeration, we included known group study designs that defined their reference standard based on criteria incorporating both clinical findings and performance on psychometric testing to classify individuals as exaggerating (within diagnostic test terminology, the target positive group), or not exaggerating (the target negative group) their symptoms (17, 18). Examples of two commonly used known group designs are the Slick, Sherman, and Iverson criteria for malingered neurocognitive dysfunction (19) and the Bianchini, Greve, & Glynn criteria for malingered pain-related disability (20)*.”**

16. Explicitly state how your study addresses gaps or contradictions found in previous studies. For example, why past standardisation efforts failed. More emphasis on the relevance and novelty of the study is essential.

**Reply 16: We undertook the first systematic review of symptom exaggeration among IME attendees. This has been explicitly stated and clarified in our introduction.**

17. While the IME concept and symptom exaggeration are introduced, there is little detail about the methodology or variables that will be assessed;
- Please articulate your hypotheses with the rationale;
- To improve flow, consider adding a sentence explaining why IMEs are prone to variability, and how symptom exaggeration may influence;
- Summarise the objectives and hypotheses clearly in one sentence.

**Reply 17: Thank you for your feedback. As noted in the reply to comments 14 we have now added the below to further clarify our objective and reasons for it:**

**“*Whether or not IME assessors consider symptom exaggeration therefore has the potential to lead to very different conclusions; however, the prevalence of exaggeration among IME attendees is uncertain and individual studies report rates as low as 18% or as high as 68%.*”**

18. Overall, there are some worrying aspects in Introduction:
The Introduction needs a clearer flow. Consider providing the background and context of IMEs, then move into the issue of symptom exaggeration. After that, it's important to debate what previous studies have found and highlight their gaps. You can then explain how this study will ease those. Concluding with the rationale, objectives, and hypotheses will give the Introduction a strong structure. Consider to mention why the study is important since it'll make more engaging and relevant for a broader audience

**Reply 18: Thank you for your feedback. We have made changes accordingly to ensure clarity and better flow as noted in our responses to comments 14 and 17.**

19. The text shifts abruptly between topics, like disability stats and symptom exaggeration, without clearly connecting them or stating the rationale. Brief mentioning the methods used to measure symptom exaggeration is really important

**Reply 19: The methods for measuring symptom exaggeration that we considered eligible are defined in the methods section under eligibility criteria as noted in our response to comment 15.**

20. The authors could provide more context to strengthen their argument. IMEs often focus too narrowly on the biomedical model, overlooking psychosocial factors and work-related conditions, which influences the fairness and consistency of evaluations. The lack of standardisation and unified guidelines contributes to these issues. Additionally, biases in IME practices, such as favouring employer-provided information over patient accounts, lead to underdiagnosis and lower disability ratings compared to treating physicians. This bias fosters distrust and adds to the inconsistencies in the IME process. For more details, see 10.1097/PEC.0000000000000487 and 10.1007/978-3-319-71906-1

**Reply 20: Thanks for this citation, which we have now included the justify the following added statement:**

**“*Independent evaluators may focus too narrowly on a biomedical model to explain symptoms, without sufficient attention to psychosocial and work-related factors***.”

21. Another aspect contributing to IME variability is the difference in approaches, prognoses, and standards among evaluators. Studies show that diagnostic discordance rates can exceed 80%, with higher discordance linked to increased mortality (OR ≈ 1.2), underscoring the need for standardised protocols. Inconsistent assessments can lead to delayed treatment, denied disability benefits, and declining patient quality of life. Standardisation would improve accuracy and consistency, reducing patient burden and enhancing trust in IMEs. Furthermore, the focus on the biomedical model overlooks the role of psychological and social factors, suggesting that adopting a biopsychosocial approach could yield more accurate evaluations

**Reply 21: In terms of the need for standardised protocols, as noted in our introduction, a recent systematic review suggested that standardization of the assessment process may improve the reliability of IMEs; however, two subsequent studies have failed to support this hypothesis (Reference: Kunz R, von Allmen DY, Marelli R, Hoffmann-Richter U, Jeger J, Mager R, et al. The reproducibility of psychiatric evaluations of work disability: two reliability and agreement studies. BMC psychiatry. 2019;19(1):1-15.). This is what led us to explore another potential source of variability in IME assessments which was the prevalence of symptom exaggeration.**

22. The Introduction mentions IMEs and symptom exaggeration, but it fails to clearly state the objectives or what the study aims to explore. Providing specific objectives and hypotheses would make it easier for readers to understand the focus of the research

**Reply 22: We have clarified this and added to the text as noted in our responses to comments 14, 17 and 18.**

23. Explain how the variability in IME outcomes could be influenced by symptom exaggeration, tying it to the broader context of disability assessments
**Reply 23:** **Thank you for your feedback. As noted in the reply to comments 14 and 17 we have now added the text underlined below to further clarify our objective and reasons for it:**

**“Another potential source of variability in IME assessments is symptom exaggeration (3). Patients referred for IMEs often present with subjective complaints (e.g., mental illness, chronic pain) and may feel pressure to emphasize their level of impairment to qualify for wage replacement benefits, receiving time off work, or other incentives (3, 9, 10). Whether or not IME assessors consider symptom exaggeration therefore has the potential to lead to very different conclusions; however, the prevalence of exaggeration among IME attendees is uncertain and individual studies report rates as low as 18% or as high as 68%. To address this gap in the literature, we undertook the first systematic review of observational studies to explore the prevalence of symptom exaggeration among IME examinees in North America.”**

24. Strengthen the rationale by incorporating previous findings on symptom exaggeration in IMEs

**Reply 24:** **Our is the first systematic review on this topic, but we have now added the range of symptom exaggeration reported by individual studies on this issue (from 18% to 68%).**

25. The study focuses on symptom exaggeration as a source of variability in IMEs, an underexplored area. While past findings checked the reliability of IMEs, this work could provide important insights on how symptom exaggeration influences assessments of disability. Please consider providing a clearer explanation of why this is relevant

**Reply 25: As noted in our responses to comments 14, 17 and 23, we added text to further clarify our hypothesis and objective.**

26: Providing more context on how symptom exaggeration might vary by condition is important; for example, variations in symptom exaggeration between conditions like traumatic brain injury and chronic pain will make the argument more cohese

**Reply 26: We did not find any subgroup effect for type of clinical condition, but we do acknowledge this issue in our Discussion section and allow that limited variability of conditions among studies eligible for our review reduced our ability to explore this issue fully:**

**“*A previous survey of 131 US board-certified neuropsychologists conducting forensic work found that, on average, they estimated symptom exaggeration occurred in around 30% of examinees claiming personal injury, disability, or workers’ compensation. However, estimated prevalence ranged considerably by diagnosis – from an average of 41% for mild head injuries to 2% for vascular dementia (39). Our review found no evidence for differences in the prevalence of symptom exaggeration based on clinical condition, but most patients among eligible studies presented with either mild TBI or chronic pain.***”

27. Please clarify the relevance of the European results - what are the similarities or differences between IME in continents? And expand this part. Consider that healthcare systems are different, especially due to different geographical frameworks

**Reply 27: We restricted our focus to North America as there may be important differences between IMEs conducted within North America where social insurance for disability is limited and Europe where social insurance is prominent. Therefore, we restricted our review to studies conducted in North America.**

28. I'd highly suggest to introduce the concept of symptom exaggeration within the context of the study

**Reply 28: Thank you. This concept is described in the introduction as follows:**

**“Another potential source of variability in IME assessments is symptom exaggeration (3). Patients referred for IMEs often present with subjective complaints (e.g., mental illness, chronic pain) and may feel pressure to emphasize their level of impairment to qualify for wage replacement benefits, receiving time off work, or other incentives (3, 9, 10).”**

29. The Introduction is brief and lacks depth to establish a clear framework. Without the mentioned components, the Introduction feels underdeveloped and won't effectively set ground for the study. This can make the interpretation challenging without a solid rationale of the study
**Reply 29: We have considered your comments and made changes accordingly to the introduction as noted previously. We believe these additions have greatly improved this section and thank the reviewer for their suggestions.**

**Methods related comments**

30. The details about PRISMA and MOOSE are not provided. For example, the authors didn't report the refs for each checklist, and didn't specify what workflow (e.g. screening, extraction, etc.) was followed. Including detailed information from both checklists, such as principal items, would enhance the presentation. The overall impression is, "How did the authors follow these guidelines?" Finally, why wasn't the systematic review registered on PROSPERO, the widely recommended registry for such studies?;

**Reply 30: We have provided the references for both PRISMA and MOOSE in the text (as references 11 and 12) as follows:**

**11. Moher D, Shamseer L, Clarke M, Ghersi D, Liberati A, Petticrew M, et al. Preferred reporting items for systematic review and meta-analysis protocols (PRISMA-P) 2015 statement. Systematic reviews. 2015;4(1):1.**

**12. Stroup DF, Berlin JA, Morton SC, Olkin I, Williamson GD, Rennie D, et al. Meta-analysis of observational studies in epidemiology: a proposal for reporting. Jama. 2000;283(15):2008-12.**

**We have now included both checklists, with details on where in our manuscript we adhered to each item, as Supplentary files.**

**As for registration of the protocol, Open science framework is a reliable and commonly used registry as well. Also, at the time of registration, PROSPERO were still in a time lag post pandemic and the large amount of COVID research beng registered. Wait times for approval were >6 months.**

31. The search strategy, while reported in the Sup. file, could be included in the main text to enhance clarity and robustness.

**Reply 31:** **Thank you for your feedback. We did not include the search strategy in the main text due to its length (3 pages). However, we have now added some details of the approach to enhance clarity:**

**“*The search strategies were developed using a validation set of known relevant articles and included a combination of MeSH headings and free text key words, such as malinger* or litigation or litigant or "insufficient effort".*”**

32. Why include studies in other languages? Are the terms and interpretations the same across different contexts?

**Reply 32:** **We expanded our search to include English, French and Spanish-language studies to reduce language bias that can result from restricting searches to English-language only. We were comprehensive in the terms we used to ensure all known synonyms were considered. These terms were reviewed by experts in this field.**

33. Please refine the verb tense for consistency.

**Reply 33:** **Thank you for this observation. We made the appropriate revisions using tracked changes in the attached manuscript.**

34. Several aspects of the design require clarification, particularly concerning the definitions, variable selection, and operationalisation. There is also a lack of explanation for the focus on specific countries. The justification for study exclusions, and the criteria for classifying symptom exaggeration could be detailed. Additionally, the handling of population overlap needs to be explained. Furthermore, if six authors conducted the screening, how was inter-rater reliability assessed? Failing to address these concerns risks making the study appear biased and unreliable

**Reply 34: Thank you for your comments. Kindly find our responses to your 4 points below:**

(1) **In terms of definitions, we ensured our eligibility is clear, definitions are provided, and justifications are made as noted in our methods section under eligibility criteria. For all included studies, the reviewers abstracted the following data: name of first author, year of publication, participant demographics, referral source(s), criteria for establishing symptom exaggeration and reference standard, and the prevalence of symptom exaggeration. We agree that details on criteria used by study authors to define symptom exaggeration are important and have provided this information (as well as the established psychometric properties of the tools) in eTable 4 in our Supplementary File.**
(2) **As for countries, we restricted our focus to North America as there may be important differences between IMEs conducted within North America where social insurance for disability is limited and Europe where social insurance is prominent.**
(3) **In our methods, we drafted a plan for when there were cases where one study published multiple iterations and there was population overlap, we would include only the study with the larger sample size. However, we did not face that issue and did not find any studies with overlapping populations. We have now added a statement to the results to reflect that.**
(4) **We agree that reliability of findings is important. However, the book by Gough, D., Oliver, S., & Thomas, J. (2017). An introduction to systematic reviews. SAGE. emphasizes that while IRR can improve the credibility of a review by providing consistency checks, it is not universally required or applied due to resource constraints and varying disciplinary standards. Instead we took multiple measures to ensure consistency starting from (1) having a predefined protocol that outlines selection criteria and procedures very clearly and training reviewers to ensure understanding of the set criteria; (2) we prefaced data abstraction with calibration exercises to optimize consistency and accuracy of extractions and continued to do calibration exercises and training sessions till all members were confident with their judgements and we noted minimal disagreement; and (3) for any disagreements that came up, we resolved them through discussion as noted in our manuscript rather than measuring IRR; (4) we had weekly meetings to check in and ensure no issues were arising and if any to resolve them directly so they don’t carry forward.**

35. The eligibility criteria lack a clear rationale for including certain types of studies while excluding others. For instance, the inclusion of studies that "assessed the prevalence of symptom exaggeration using a known group design or multi-modal determination method" seems arbitrary without explaining these specific designs (c.f. 10.1212/CPJ.0000000000000092; and why they are superior or necessary for the systematic review. Please elaborate on why these aspects were chosen.

**Reply 35: Thank you. We provided a detailed explanation in the manuscript under our eligibility criteria as to why we chose to only include known group designs and why the other types of studies were deemed inappropriate. The text in the manuscript under study selection reads as follows:**

**“*…assessed the prevalence of symptom exaggeration using a known group design or multi-modal determination method (15, 16). As there is no singular reliable and valid criteria (reference standard) in the literature that is used to assess for symptom exaggeration, we included known group study designs that defined their reference standard based on criteria incorporating both clinical findings and performance on psychometric testing to classify individuals as exaggerating (within diagnostic test terminology, the target positive group), or not exaggerating (the target negative group) their symptoms (17, 18). Examples of two commonly used known group designs are the Slick, Sherman, and Iverson criteria for malingered neurocognitive dysfunction (19) and the Bianchini, Greve, & Glynn criteria for malingered pain-related disability (20). We excluded studies that used only beyond-chance scores on symptom validity tests as an indicator of symptom exaggeration, since beyond-chance scores are infrequent and likely to result in underestimates (21, 22)*.”**

36. If there is no reliable and valid standard for assessing symptoms, it's somewhat concerning to understand the reliability of the categorisation of the quality of the included studies. Actually, some studies report strategies for a reliable assessment of symptom exaggeration. Consider mentioning symptom validity tests. I think providing a detailed assessment criterion or more than one tool would be important (c.f. 10.1002/nur.10092). Consider using the Cochrane risk of bias or other tools (c.f. 10.1136/bmj.d5928; 10.1016/j.acn.2005.02.002). Also, explain how the authors ensured the reliability of the findings and what kind of psychometric assessments were performed (c.f. 10.1007/s12207-021-09436-8; 10.1016/j.acn.2005.02.002);

**Reply 36: As mentioned in our manuscript, none of the known group designs used to evaluate symptom exaggeration have undergone reliability and validity testing. We searched the literature to assess whether the forced-choice tests used in these criteria demonstrated adequate psychometric properties, which they did as noted in e-table 4 in our Supplementary material. However, in our discussion we highlight the urgent need to evaluate in future research the inter-rater reliability of known group systems to appraise symptom exaggeration. Validation of such assessment systems is extremely challenging, but indirect evidence of validity could be acquired by evaluating accuracy in distinguishing between volunteers who were or were not exaggerating symptoms. These efforts are outside the scope of our study but are research priorities and gaps that should be addressed in the future.**

**We used the GRADE approach to assess our certainty in the evidence that included an overall risk of bias assessment across studies, and ratings on inconsistency, indirectness, imprecision and publication bias. This thorough assessment led us to a low certainty rating which reflects the concerns of our team and that of the reviewers in a transparent way and highlights the need for future research to provide a better understanding into this phenomenon and explore these findings further using rigorous study designs.**

**In terms of our risk of bias assessment tool, the Cochrane tool is only applicable to randomized controlled trials which were not eligible for our review. We therefore worked with risk of bias experts (e.g., Gordon Guyatt) to develop risk of bias items that are important and relevant for known group study designs (see eTable 2).**

37. The preference for including studies with larger sample size when population overlap exists introduces a potential for selection bias. Larger sample sizes do not always equate to better quality for meta-analysis. It'd be interesting to refer to relevant literature on this matter (e.g. 10.1002/sim.1186; 10.1136/bmj.d7762). I'd highly suggest conducting robust sensitivity analyses to assess the influence of including or not certain studies (c.f. 10.1016/j.jclinepi.2004.01.018; 10.1111/rssc.12440);

**Reply 37: Thank you for your comment. As noted in our response to comment 34, in our methods section we drafted a plan for when there were cases where one study published multiple iterations and there was population overlap, we would include only the study with the larger sample size. However, we did not face that issue and did not find any studies with overlapping populations thus we have no concerns around selection bias due to this issue. We added a statement in the results section to clarify that.**

**Results related comments**

38. The authors could consider some approaches: a. run both fixed- and random-effects and scrutinise if there are substantial differences in the effects (i.e. if found, heterogeneity would be a really worrying aspect), b. identify studies with a high risk of bias, or small sample, remove them, and conduct sensitivity analysis (e.g. using l-o-o or cumulative sensitivity analysis). Then check the results, specifically evaluating the existence of differences in effects, CIs overlap, and changes in I² values. This would also be important when analysing subgroups

**Reply 38: Thank you for your comment.**

1) **In terms of fixed versus random effect: fixed effect models are only appropriate when there is homogeneity which is not the case here as we are aware heterogeneity exists in the study population.**
2) **In terms of small study effect, the funnel plot particularly in the small study section at the bottom is symmetrical and our Eggers test showed no evidence of small study effects (eFigure 2, p=0.13).**
3) **In terms of sensitivity/subgroup analyses we did explore the impact of risk of bias on our findings:**

**“*We assessed variability between studies based on three a priori hypotheses. We assumed a higher prevalence of symptom exaggeration with: (1) greater strength of the reference standard, and (2) higher risk of bias on a component-by-component basis. We also explored for subgroup effects based on type of clinical condition but did not pre-specify an anticipated direction of association. We conducted subgroup analyses if there were two or more studies in each subgroup, and evaluated credibility of significant subgroup effects using ICEMAN criteria (27). We performed meta-regression to explore the relationship between proportion of female sex, mild severity, mean age, and education years with the prevalence of symptom exaggeration during IMEs. If the meta-regression was statistically significant, we used visual inspection of the associated scatterplot to estimate a threshold and conducted subgroup analysis*.”**

39. While beyond-chance scores are rare, they can still provide valuable insights into clinical findings (c.f. 10.1016/j.spinee.2004.11.016; 10.1186/s12874-016-0108-4). For instance, some studies have employed complementary approaches to assess symptom exaggeration (c.f. 10.1097/00001199-200004000-00006; 10.1007/BF01874896); Please elaborate;

**Reply 39: We agree that beyond-chance scores are valuable, but on their own they are likely to underestimate symptom exaggeration due to the fact that beyond-chance scores primarily measure agreement beyond what’s expected by random chance. They are not designed to detect systematic response biases. As such, we included studies that assessed the prevalence of symptom exaggeration using a known group design or multi-modal determination method. As there is no singular reliable and valid criteria (reference standard) in the literature that is used to assess for symptom exaggeration, we included known group study designs that defined their reference standard based on criteria incorporating both clinical findings and performance on psychometric testing (beyond-chance scores) to classify individuals as exaggerating or not exaggerating their symptoms. This approach is consistent with the articles cited by the reviewer. For example, Iverson and Binder (2000) conclude:**

**“The assessment process for the purpose of ruling out malingering includes several steps. First, the clinician should consider the severity of the injury. For example, as noted previously, mild head injuries are typically not associated with severe deficits, not even in the forensic arena. In contrast, one is not surprised at severe deficits if a patient was comatose for a month and the brain scans showed abnormalities. Second, careful review of the medical record is necessary to look for inconsistencies such as nonorganic abnormalities on orthopedic or neurologic exams or between such examinations. The record also should be examined for inconsistencies regarding social and medical history. For example, some patients may exaggerate their military or educational records or deny or minimize previous psychiatric history. Third, testing should include both a thorough neuropsychological assessment and testing for biased responding, with additional tests of emotional functioning as needed.”**

**References:**

- **Rogers, R. (2008). Clinical Assessment of Malingering and Deception.**
- **Aguerrevere LE, Greve KW, Bianchini KJ, Ord JS. Classification accuracy of the Millon Clinical Multiaxial Inventory–III modifier indices in the detection of malingering in traumatic brain injury. Journal of Clinical and Experimental Neuropsychology. 2011;33(5):497-504.**
- **Cook RJ, Farewell VT. Conditional inference for subject‐specific and marginal agreement: two families of agreement measures. Canadian Journal of Statistics. 1995;23(4):333-44.**

40. In studies where symptom exaggeration is being evaluated, the lack of blinding could significantly influence the results. Therefore, I would highly encourage the authors to expand this part;

**Reply 40: Blinding is an essential safeguard for protecting the integrity of randomized trials; however, in studies eligible for our review, which were observational, clinicians assessed real claimants for the possibility of symptom exaggeration. We did not include studies that used volunteers – some of whom were advised to exaggerate their symptoms and some who were not. As such, the status of a claimant (exaggerating vs. not) was not known prior to assessment, and so blinding was not applicable.**

**We are unaware of any studies that have used deception to blind patients attending for an IME, and suspect that ethical considerations would preclude such a study from proceeding. Further, patients who are unaware that they are attending for an independent medical evaluation, whose role is, in part, to establish the validity of their impairment, would likely behave in a systematically different manner as they would when knowingly attending an IME. Thus, the results would lack validity.**

41. The databases used in the search are comprehensive. However, expanding the search to include other databases, such as Scopus, could ensure that no additional relevant refs are missed. Could the authors clarify why Scopus or similar databases were not included in the search strategy?

**Reply 41: It is important to draft a search that balances between thoroughness and efficiency. We chose not to include searching in Scopus as we felt doing so would have added more work for little return. This is because most of Embase’s journals are included in Scopus, and Scopus does not include the granular Emtree indexing that Embase is known for. As a safeguard against missing any relevant studies, in addition to the four electronic databases we searched, we also screened the reference lists of all included studies for additional eligible articles.**

42. Additionally, more details regarding the filters applied during the search would be essential. For instance, were specific study designs or types prioritised during the screening process, and if so, which ones?

**Reply 42:** **We did not include any filters in our searches in order to maximize sensitivity, and we have now clarified this in our Methods section.**

43. As recommended by Cochrane, systematic reviews should be updated to maintain their relevance and accuracy. Could the authors provide an approximate timeline for when they plan to refresh the search.

**Reply 43: We agree. Since the speed of publishing is slower in this field compared to others, we suggest updating this review in the next 5 years. We added a section in the discussion under implications for research and practice to reflect this. It now reads as follows:**

**“Systematic review update**

**Considering the speed at which studies exploring the prevalence of symptom exaggeration among IME attendees are published, we suggest this review be updated within the next five years.”**

44. How were duplicates handled during the search process?

**Reply 44: In our PRISMA flow chart, we noted the duplicates that were identified when uploading the citations for screening in the identification section while those found during screening were reported under the subsection ‘duplicate/overlapping publication’ in the box titled: “full text articles excluded’.**

45. The search strategy is well-constructed and covers several important terms across databases. However, there are some concerning aspects. The use of broad terms such as 'validity' and 'disability' may result in many extraneous results, diminishing the relevance of the retrieved studies and also increasing the overall screening effort. Additionally, applying filters (e.g. specific diagnostic criteria or populations) could help enhance the reliability and focus on more relevant studies. Please clarify the rationale for not using such refinements.

**Reply 45: We worked with a senior medical librarian and experts in the field to develop a search that attempted to balance thoroughness with efficiency. We used broader terms to ensure we captured all potential studies, but this did not increase our number of hits tremendously and remained manageable for screening. Also, we did not use study design filters as this area may not be indexed in a similar way and not all known group design studies are labelled as such. Therefore, we avoided search filters to minimize the risk of missing eligible studies. We do agree that further optimization of our search is likely possible.**

46. Please clarify the relevance of the transformation using F-T arcsine in your study. This is really important based on your design. The concern is to provide an explanation of parameters and the rational, enhancing clarity for readers outside the field

**Reply 46: Thank you. The prevalence data usually follows a binomial distribution. When the sample size is small or the proportion gets closer to the margins (0% or 100%), the Wald‐type confidence intervals (CIs) of the prevalence may be outside the range of 0% to 100%, which is non-sensical. To further clarify this in the manuscript we added the following text along with references:**

**“*We used a random-effects model to pool data for the prevalence of symptom exaggeration among IME examinees, and used a Freeman-Tukey double arcsine transformation to stabilize the variance (1,2). This transformation avoids producing confidence intervals (CIs) that include values lower than 0% or greater than 100% (1,2). We used the DerSimonian and Laird method (3) to pool the estimates based on the transformed values and their variances, and then the harmonic mean of sample sizes for the back‐transformation to the original units of proportions (4).*”**

**References:**

**1. Freeman MF, Tukey JW. Transformations Related to the Angular and the Square Root. The Annals of Mathematical Statistics. 1950;21(4):607-611.**

**2. Nyaga VN, Arbyn M, Aerts M. Metaprop: a Stata command to perform meta-analysis of binomial data. Arch Public Health. 2014;72(1):39. doi:10.1186/2049-3258-72-39**

**3. DerSimonian R, Laird N. Meta-analysis in clinical trials. Control Clin Trials. Sep 1986;7(3):177-88. doi:10.1016/0197-2456(86)90046-2**

**4. Miller J. The Inverse of the Freeman – Tukey Double Arcsine Transformation. Am Stat. 1978;32(4):138.**

47. The trim-and-fill method could provide a more accurate adjustment for potential bias. Overall, after assessing any asymmetry in the funnel plot, this trims and fills missing data points to 'reach' symmetry, recalculating the overall effect size. Could the authors consider applying this approach and debating the influence on their analysis?

**Reply 47: We agree that this methodology is appropriate when small study effects are detected; however, we did not find any evidence of small study effects through Egger’s test or our funnel plot.**

48. Please consider expanding the analyses by assessing Tau² (i.e. between-study variance), which could provide better insights into heterogeneity (c.f. 10.1136/bmj.327.7414.557);

**Reply 48: Thank you for your feedback. We agree with the reviewer and have now calculated Tau^2^ and added it in Table 2 and to all our forest plots.**

49: When conducting stats analyses (e.g. meta-regression, subgroup analyses), it’s essential to be cautious of small-study effects, which can influence the robustness of your findings

**Reply 49: We agree, however, neither our funnel plot nor Egger’s test showed evidence of small study effects.**

50. The Results are intriguing. However, the observed high variability and low certainty, as well as the association between women and higher symptom exaggeration, raise concerns about potential confounding factors. Differences in populations, diagnostic tools, and study designs likely contribute to this variability. The authors are encouraged to explore these issues more thoroughly, perhaps expanding the meta-regression to include more DVs for multivariate analysis. Consider additional approaches like structural equation modeling to reduce the number of variables, along with PCA and clustering. This would enhance the depth of your analyses, results, and overall presentation

**Reply 50: We assessed any confounding factor that was reported in the original studies, and we considered conducting a multivariable meta-regression if there were two or more significant confounders identified. However, since we only found one significant factor from those we could test (i.e. proportion of females in studies) we did not have enough significant factors to conduct a multivariate analysis.**

**We have now added under our ‘Strengths and Limitations’ section another limitation of individual studies regarding lack of stratification of symptom exaggeration by subgroups. It now reads as:**

**“*Another limitation of the primary studies is the lack of stratification of prevalence of symptom exaggeration according to possible effect modifiers, such as sex. Doing so would facilitate within-study subgroup analysis, which are less subject to confounding*.”**

51. Please specify test parameters (e.g. effect sizes, confidence intervals) alongside p-values for a more comprehensive understanding of the results

**Reply 51: We reviewed our results and discussion and added test parameters where they were missing.**

52. Ensure consistency in decimal places throughout the results section;
**Reply 52: Thank you. We reviewed the results section to ensure consistency.**

53. The CIs for symptom exaggeration in women should be carefully examined, as overlapping CIs could indicate heterogeneity or residual variance;
**Reply 53: Unfortunately, due to the lack of reporting of symptom exaggeration data by sex we were unable to conduct a subgroup analysis to compare groups. However, we were able to assess studies with a higher versus lower proportion of women and found a potentially credible subgroup effect. Thus, when assessing our rating for inconsistency, we rated down by one level due to heterogeneity (wide range in prevalences) which was only partially explained by sex. Judging the extent of heterogeneity was based in accordance with GRADE guidance (**[**https://www.sciencedirect.com/science/article/abs/pii/S089543561100182X**](https://www.sciencedirect.com/science/article/abs/pii/S089543561100182X)**).**

54. Given these shortcomings, I suggest the authors: a) Check for studies with small samples or wide CIs and identify possible outliers, b) Carefully consider the influence of outliers and residuals, c) Reporting I² and Tau² stats to assess heterogeneity. If heterogeneity is high, outliers could be driving the variation between studies

**Reply 54: Thank you once again for this very helpful feedback. We have made changes as noted in the previous comments to address these issues.**

**Discussion related comments**

55. The Discussion is well-structured but lacks conciseness, particularly in the “Relation to Other Studies” and “Implications for Future Research” sections. Streamlining these parts would enhance clarity and flow

**Reply 55: Thank you for your comment. We understand and agree with the value of being concise and have attempted to revise our Discussion section with this in mind.**

56. While the study finds low-certainty evidence of symptom exaggeration in IMEs, the authors didn't provide sufficient arguments about what drives this low certainty. The sources of uncertainty (e.g., inconsistency, bias, imprecision) could be more clearly outlined. Also, consider debating the influence of heterogeneity

**Reply 56: Thank you. We have now clearly stated the rationale for downgrading to Low certainty of evidence in the first paragraph of our Discussion section, and highlighted that female sex may be a source of heterogeneity:**

**“*Our systematic review and meta-analysis of observational studies found low certainty evidence, rated down due to risk of bias and imprecision, that symptom exaggeration may be common among individuals attending for IMEs in North American studies, affecting approximately 1 in 3 assessments. The prevalence of symptom exaggeration was very similar across different clinical conditions and was higher in studies that enrolled a larger proportion of female attendees (47%, 95%CI 36 to 58) vs. a lower proportion of female attendees (31%, 95%CI 28 to 35; test of interaction p=0.02) (Figure 2, Table 2, e-Table 3).*”**

57. Some examples of how different studies operationalised symptom exaggeration differently and how this could be 'solved' seem a very interesting approach;
**Reply 57: We agree this is a critical issue, and we attempted to address assessment method as a source of heterogeneity in two ways: (1) by restricting eligible studies to only those that used a known group or multi-modal approach to determining exaggeration, and (2) conducting a subgroup analysis based on the credibility of their reference standard. However, no known-group method has been validated, and our subgroup analysis may have been non-significant due to limited variability. We have highlighted both of these limitations and called for further research to address these issues.**

58. Suggest practical steps for developing and validating new assessment tools for IMEs;
**Reply 58: We do note under research priorities that an important area of work is the development and validation of a structured and comprehensive approach to identify symptom exaggeration in IME assessments. We also touch on what should be included or considered in this approach (i.e. observed versus reported abilities, findings of other providers, self-reported history that is discrepant with documented history, and administration of symptom validity tests.). However, detailed guidance for how to develop and validate new tools for IMEs is outside the scope of our review.**

59. Additionally, scrutinise CIs more carefully for potential overlap, as this could indicate heterogeneity;
**Reply 59: We explored reasons for heterogeneity to the extent that the reported data in the primary studies allowed. Accordingly, as noted previously we were only able to partially explain this heterogeneity based on the proportion of females enrolled into studies. We then made transparent ratings about the extent of unexplained heterogeneity and how that impacted our certainty in the evidence based in accordance with GRADE guidelines number 7 (**[**https://www.sciencedirect.com/science/article/abs/pii/S089543561100182X**](https://www.sciencedirect.com/science/article/abs/pii/S089543561100182X)**).**

60. There is a concerning aspect regarding the connection to other findings. The arguments lack depth and focus mainly on 'correlation' with somatic symptoms, which may not fully align with IMEs or the study's objectives. Clarify the main differences between studies, focussing on the authors. The link between gender and symptom exaggeration could be simplified, and the data more sharply refined;
**Reply 60: Our material on related studies explored perceived prevalence of symptom exaggeration vs. what our review found, possible explanations for the subgroup effect we found based on sex, and whether professional organizations provide clear guidance on how assessors should explore for symptom exaggeration (they do not). We feel this is a reasonable approach but would be happy to introduce new material as suggested.**

**We attempted to explore how differences among studies might contribute to heterogeneity in our findings (e.g., risk of bias, proportion of women, credibility of the reference standard used for their assessment of exaggeration), which was partially successful. We are uncertain of what the reviewer is suggesting in regard to “focussing on the authors”.**

**Our section on the possible association between sex and symptom exaggeration is currently four sentences, which we feel is concise; however, we would be happy to consider specific suggestions to refine this section further.**

61. Please provide a detailed explanation of how the multimethod approaches varied across studies and how the differences may have influenced the outcomes of the meta-analysis;
**Reply 61: Thank you for your comment. We only included studies that used reference standards based on criteria incorporating both clinical findings and performance on psychometric testing to classify individuals as exaggerating (within diagnostic test terminology, the target positive group), or not exaggerating (the target negative group) their symptoms. We explored if our confidence in the reference standard may have impacted the results and did not find a credible subgroup effect; however, we also highlight in our limitations section that our results may have been affected by limited variability (i.e., the large majority of reference standards were judged of “moderate” confidence).**

62. The limitations are mentioned, but other biases, such as cultural or healthcare factors, could be potential confounders. Also, the lack of standardization and the absence of reliable assessments should be addressed;
**Reply 62: We have now added to the text that the generalizability to other contexts (cultural or other health care factors etc.) is uncertain and it now reads as follows:**

**“..the generalizability of our findings to other jurisdictions, contexts, and clinical conditions, is uncertain.”**

**As for the lack of standardization and the absence of reliable assessments we noted this as a limitation and in our section on implications for future research. Text from our manuscript reads as follows:**

**“*Another limitation of our review is the absence of a compelling reference standard for symptom exaggeration. Furthermore, even within the same reference standard, operationalization can be variable, which affects prevalence. Also, a major limitation of the current evidence is that none of the known group approaches for evaluating symptom exaggeration have undergone reliability and validity testing*.”**

**“*An urgent research priority is evaluating the inter-rater reliability of known group systems to appraise symptom exaggeration. Validation of such assessment systems is extremely challenging, but indirect evidence of validity could be acquired by evaluating accuracy in distinguishing between volunteers who were or were not exaggerating symptoms. Another research priority is the development and validation of a structured and comprehensive approach to identify symptom exaggeration in IME assessments…”***

63. The link between Results and the Discussion could be clearer. Highlighting specific findings from the results, such as effect sizes or CIs, would enhance the reliability and make the conclusions more solid;
**Reply 63: Thank you for your feedback. We added the effect sizes and CIs in the first paragraph of the discussion that reiterates our main findings to enhance clarity.**

64. The terminology distinguishing symptom exaggeration from malingering is unclear. Please clarify this distinction;
**Reply 64: We noticed that most studies use the terms interchangeably and often label their criteria as criteria to assess malingering. However, when using the term malingering one presumes intent. Thus we felt it was an important suggestion to note in our discussion that considering how ‘clinicians are, understandably and appropriately, hesitant to assign a label of malingering…. (45)’ the term symptom exaggeration may be a more appropriate replacement.**

**Tables and illustrations related comments**
65. The search strategy appears to include terms that may retrieve numerous irrelevant studies. The authors could consider refining the search by removing broad terms and avoiding overuse of wildcards. This can reduce the retrieval of unnecessary results and save future researchers from conducting an excessively lengthy and unfocused search. For example, terms like "independent" and linked terms such as "MMPI" and "work capacity" could be refined or excluded to enhance specificity. In using the current strategy, I encountered studies from adjacent fields, such as business or diagnostics for conditions unrelated to the focus of the review;
**Reply 65: As noted previously in our response to comment 45, we worked with a senior medical librarian and experts in the field to develop a search that balances thoroughness with efficiency. We used broader terms to ensure we captured all potential studies, but this did not increase our number of hits tremendously and remained very manageable for us to screen through.**

66. The risk of bias Table lacks clarity and cohesion, as the studies are not organized alphabetically or by any discernible pattern, making it hard to follow. The use of consecutive sampling raises concerns about potential selection bias, which could influence the outcomes and, consequently, generalisation. Additionally, the 5% cutoff for missing data, though comprehensive, should be supported by relevant evidence to justify. While age and education are important demographics, it's unclear why other variables, such as gender, were not included. Expanding the demographic could provide a more comprehensive understanding of their influence on the findings;
**Reply 66: Thank you. We have revised the risk of bias table in alphabetical order to enhance clarity.**

**Consecutive sampling, which was the method used in all our included studies, involves selecting every eligible individual who meets the study criteria until the required sample size is reached. This ensures that all eligible participants who are available within a specified timeframe are included. Consecutive sampling reduces the risk of selection bias associated with non probability sampling approaches such as convenient sampling thus providing data that is more generalizable to the specific population being studied and improving the external validity within that population.**

**As for the 5% missing data. That was not a cutoff but the amount of missing data in studies. As reported in our manuscript we considered >20% missing data to be concerning as this is a commonly used cutoff in systematic reviews. All studies had <5% missing data and thus were not considered at high risk of bias with regards to that domain. We have clarified this in our table footnotes to ensure there is no confusion to the reader.**

**In terms of gender, no study reported the results for symptom exaggeration for men and women separately. We have now highlighted this as a limitation. we did conducted a between study subgroup analysis to explore whether proportion of females affected the prevalence of IME, and as our subgroup analysis was significant (p=0.02) we reported our findings according to these subgroups.**

67. The Table on psychometric data raises concerns as it presents tests used in completely different settings, likely increasing heterogeneity. The variability in sample sizes, demographics, and the use of unreliable tools diminishes the potential for generalisation of the findings. Additionally, there are several outdated refs that should be updated to reflect current practices. Lastly, please double-check the grammar, as there are issues with punctuation and formatting that need correction

**Reply 67: We noted in our methods under risk of bias assessment that, in addition to the other considerations we assessed, we evaluated whether the criteria for establishing symptom exaggeration and forced-choice tests used in these criteria were reliable and valid. We did not find any studies that assessed the reliability and validity of the overall criteria used for assigning symptom exaggeration, but we did find studies evaluating psychometric properties of the forced-choice tests used in these criteria (e-table 4). We do not maintain that this sufficient, and we rated down the certainty of evidence for our estimates of symptom exaggeration prevalence as a result of insufficient validation of all symptom exaggeration criteria. The information in e-table 4 is simply presented to give readers a sense of what evaluation of psychometric properties has been conducted on forced-choice tests we identified. We agree that some of the articles evaluating psychometric properties are older but have presented their findings to give readers a sense of what has been done in this area.**

**We are agreeable to removing this e-table if the reviewer feels it is unhelpful.**

68. It's essential to note that the funnel plot is asymmetric, indicating heterogeneity. This reinforces the importance of using the trim-and-fill method to ensure robustness. The authors could also check carefully the sensitivity analysis and assess how the funnel looks after refinement. At a glance, there seem to be small-study effects, given the number of studies on left side

**Reply 68: Eggers test did not support a finding of small study effects (p=0.13).**

69. The graph on meta-regression exhibits dispersion and variability that isn't related to demographics (i.e. as indicated by the regression line). Please implement additional metrics like CIs or residuals for clarity and include important stats like the p-values, I², and other important metrics

**Reply 69: Thanks for your comment. We have added all metrics under the meta-regression graphs to enhance clarity.**

70. The Table on study characteristics is missing important variables, like the ratio or percentage of women, including incorrect reporting (e.g. sampling being 'unclear'), and utilises confusing criteria for assessing symptom exaggeration. Please consider restructuring the Table to enhance clarity

**Reply 70: Thank-you for these comments. We have included the % of women in Table 1, replaced “unclear” sampling method with “not reported”, and revised the criteria to improve clarity.**

71. Overall, this is a well-conducted study, and I commend the endeavour. The findings presented are of notable importance. While this might sound lengthy, it is provided with the best intentions, aiming to align with the high standards expected in scientific communication.

While I found this study to be promising and well-conceived, I believe its current state may require extensive rounds, which could complicate the review process. To help streamline, I’ve provided some suggestions for the authors to consider. I encourage the authors to consider or check these and choose how they wish to proceed. Should they choose to resubmit after corrections, I'd be happy to read the ms again. I apologise if this isn’t the most positive news, but I see great potential in this work.

**Reply 71: We appreciate the time given and detailed attention to our study by the editors and reviewers which has greatly improved our manuscript.**

**Reviewer Number 1**

**General Comments:**

I read your work with great interest, and I am pleased to congratulate you on your contribution to scientific research.
I believe that the article is novel and interesting, that it has a sufficient impact, and that it adds to the knowledge base. Plagiarism was not detected. The study appears to follow relevant guidelines and provides an original contribution to the existing scientific literature. There are no flaws in the data presented, and there are no misleading or false conclusions.
The current study is scientifically valid. The applied methodology is adequate. The reasons for performing the study are clear. I recommend the article for publication in PLOS ONE in its current form.

**Reply 1 (reviewer 1): Thank you for your feedback and for taking the time to review our manuscript.**

We thank you for your time and remain hopeful that our study in its revised form will be accepted for publication in *PLOS ONE*. We look forward to hearing from you.

Sincerely,

Andrea Darzi and Jason Busse; on behalf of all authors
