# Supplementary material for: Prevalence of symptom exaggeration among North American independent medical evaluation examinees: A systematic review of observational studies
Source: PLoS One. 2025 Jun 25;20(6):e0324684. doi: 10.1371/journal.pone.0324684 (PMC12193048; doi:10.1371/journal.pone.0324684)
Supplement: Attachment — Submitted filename: IME_Response to reviewers_April 2_2025.docx [file pone.0324684.s017.docx]

April 2, 2025

RE: PONE-D-24-31136R1

Dear Dr. Fernandes,

We have received and reviewed the editors’ and reviewers’ comments on our manuscript entitled: "*Prevalence of Symptom Exaggeration Among North American Independent Medical Evaluation Examinees: A systematic review of observational studies*".

We are very grateful for the constructive comments and suggestions from the reviewers, which have improved the paper considerably. Please find our detailed responses to their comments below.

**Reviewer 2 Comments**

1. Symptom overreporting may have multiple causes (see Merckelbach et al., 2019). Symptom exaggeration due to external incentives is related to malingering. Your reply to point 64 states: "We noticed that most studies use the terms interchangeably and often label their criteria as criteria to assess malingering. However, when using the term malingering one presumes intent. Thus we felt it was an important suggestion to note in our discussion that considering how ‘clinicians are, understandably and appropriately, hesitant to assign a label of malingering…. (45)’ the term symptom exaggeration may be a more appropriate replacement." Indeed, malingering equals symptom overreporting, but symptom overreporting does not necessarily mean malingering. In the context of IME, malingering might be more obvious to use for the manuscript, though in clinical practice one should not always assume but rather take malingering into account.

**Reply 2: Thank you for raising this important point regarding the distinction between malingering and symptom exaggeration or overreporting. In our review, we avoided the term malingering because it presupposes evidence of intent, which the included studies did not investigate. None of the primary sources explicitly assessed whether participants deliberately feigned symptoms, so we adopted the more neutral phrase symptom exaggeration to describe the findings without attributing conscious deception. We trust that this explanation provides a clearer rationale for our choice of terminology. We added the following material to the background:**

**“*Also, terminology such as exaggeration, malingering, or over-reporting are defined inconsistently across studies, making it difficult to distinguish intentional deception from psychological amplification of distress.*”**

3. Both the introduction and discussion are very short, relating to the point 29 raised in the previous round. In my opinion, these comments are not sufficiently dealt with. Also the transitions between different topics within the Introduction and Discussion could be improved.

**Reply 3: We expanded and revised our introduction and discussion sections and reviewed both to improve transitions.**

4. It is stated that French and Spanish studies were included to reduce language bias, but what about culture bias? You state this later on, but this is something that could be taken into account at an earlier level.

**Reply 4: Thank you for raising the issue of culture bias. Although we included studies in English, French, and Spanish to reduce language bias, we acknowledge that restricting our review to a North American setting does not fully address cultural variability. Indeed, cultural factors (e.g., varying attitudes toward disability) may influence how patients report symptoms or interact with examiners. This may have an effect on both the prevalence of symptom exaggeration and assessors’ interpretations. Our review did not find studies addressing this issue specifically within our inclusion criteria; therefore, we could not explore these possible differences. We agree that future research should investigate how cultural diversity affects IME outcomes, with attention to factors such as language barriers, health beliefs, and potential biases among both examinees and examiners. We have now clarified in the discussion section that this is a limitation of the included studies (and therefore our review), and highlighted exploration of the impact of cultural bias on IME findings for future research.**

5. Risk of bias assessment: also relating to point 36> why were no tools used to assess risk of bias? E.g., Cochrane's? Also, what was the measure of agreement (e.g., Kappa)? The selection seems very subjective.

**Reply 5: Studies eligible for our review were observational in design, and thus the Cochrane tool (designed for Randomized Controlled Trials) was not appropriate. Instead, we collaborated with content experts and methodologists to develop key criteria specific to known group designs (e.g., sample representativeness, reference standard confidence, and missing data). After piloting these criteria, reviewers underwent several rounds of training and independently and in duplicate assessed risk of bias, resolving disagreements by consensus or a third reviewer. Although we did not calculate a kappa statistic, we have clarified these steps in our Methods to illustrate how we increased reliability for our risk-of-bias judgments.**

6. Table 2: what do the levels of inconsistency mean, especially 'serious inconsistency'? How does this relate to your conclusions?

**Reply 6: In GRADE terminology, “inconsistency” refers to the variation in results across studies (ie, heterogeneity). When GRADE tables state “serious inconsistency,” it means there was substantial variability in effect estimates that we could not adequately explain (for example, no clear reasons for differing results emerged in our subgroup analyses). Unexplained between-study inconsistency lowers our confidence in the pooled estimate, which is reflected in our GRADE assessment of the certainty of evidence. We have expanded on this in a footnote under Table 2.**

7. It is concluded that the review found no evidence for differences in the prevalence of symptom exaggeration based on clinical condition, but most patients among studies eligible for our review presented with either mild TBI or chronic pain'. This was not a (preregistered) research question, and how to conclude this based on only two clinical conditions? Other clinical diagnoses might have symptom exaggeration, but are not included in the current review.

**Reply 7: We agree. Although our review did not detect differences in the prevalence of symptom exaggeration across clinical conditions, most included studies recruited individuals with mild TBI or chronic pain. Because few other clinical populations were studied, we cannot draw definitive conclusions for other diagnoses. We have included language in the results (prevalence of symptom exaggeration and additional analyses section) and strengths and limitations section of our review clarifying the populations that were represented and highlighting that there are other patient groups that were underrepresented or absent in our review and where more research is needed.**

***“We found no significant subgroup effects for type of clinical condition (mild TBI versus chronic pain versus other conditions)…”***

***“In terms of limitations, we restricted our review to IMEs conducted in North America and eligible studies focused mainly on chronic pain and TBI. The generalizability of our findings to other jurisdictions, contexts, and clinical conditions, is uncertain.”***

8. Some reasoning errors in conclusions; if women report higher rates of symptom exaggeration, then it is logically that studies that included more women show higher raters.

**Reply 8: We anticipated that studies eligible for our review would report a range regarding the prevalence of symptom exaggeration among individuals presenting for independent medical evaluations. Accordingly, we conducted several subgroup analyses to see if we could identify one or more factors that helped explain between-study variability. Only one of our subgroup analyses, the proportion of female participants, showed a credible subgroup effect. This result supported the reporting of separate rates of symptom exaggeration between men and women.**

9. There are still some punctuation errors (extra comma use, delete space) throughout the manuscript. At times the phrasing (e.g., p.23 line 72 ('Such concerns...) could be improved.

**Reply 9: Thank you. We have reviewed the manuscript more closely and corrected any errors.**

10. For research transparency, what were the exact key words? I know they are stated in supplemental materials and that the apparently take 3 pages to explain, but I do believe these could be stated clearly in the manuscript.

**Reply 10: We have added keywords used in our literature search to the manuscript, and it now reads as:**

***The search strategies were developed using a validation set of known relevant articles and included a combination of MeSH headings and free text key words, such as malinger* or litigation or litigant or "insufficient effort" and “independent medical examination” or “independent medical evaluation” or “disability” or “classification accuracy”.***

**Review 3 Comments**

11. Dear authors of the article, I want to thank you for a well-prepared article.
The undoubted advantages of the article are: The text of the article is logically structured and understandable to the reader. The language meets the level and requirements of the scientific language. The article uses modern data analysis, adequate to the purpose of the study. The research is of high importance for science and practice. The authors openly and adequately note in the article limitations, implications for future research and practice.
I certainly recommend this article for publication in the journal, but I have some questions and recommendations for improvement.

**Reply 11: Thank you. We appreciate you taking the time to review our manuscript and address your questions below.**

12. Why were only these databases CINAHL, EMBASE, MEDLINE and PsycINFO used for analysis?

**Reply 12: We selected these four databases (CINAHL, EMBASE, MEDLINE, and PsycINFO) because they collectively capture the key biomedical, nursing/allied health, and psychological/psychiatric literature most relevant to our review topic. Also, we evaluated, with the help of a senior medical librarian, a validation set of known eligible studies to confirm our search strategy and database coverage. When our search retrieved all studies in our validation set, we were reassured that our choice of databases and search approach was sufficiently comprehensive to capture the relevant literature in this area. We further supplemented our search by scanning the reference lists of included articles, and having experts review the list of included studies, providing additional checks for completeness.**

13. In the discussion section, it is necessary to go beyond America and compare scientific data with other countries and continents

**Reply 13: Thank you for your suggestion. We have now added to the discussion section data comparing prevalence of symptom exaggeration beyond America. The text reads as such:**

***“Although our review focused on IMEs in North America, data from other regions also suggest high rates of symptom exaggeration. An observational study in Spain reported that of 1,003 participants (61.5% female), drawn from unselected undergraduates, advanced psychology students, the general population, forensic psychologists, and forensic/legal medicine physicians, one-third reported having feigned symptoms or illness (44). Data from Germany and the Netherlands suggest that one‐fifth to one‐third of clients in forensic or insurance contexts exhibit symptom overreporting (45). Further, a Swiss study found that 28% to 34% of individuals undergoing medico‐legal evaluations demonstrated probable or definite symptom exaggeration (46).”***

14. At the end of the paragraph "Eligible studies", clarify what does "large sample size" mean (what is the sample size in numbers)?

**Reply 14: Regarding this sentence: “*In cases where multiple studies had population overlap, we included only the study with the larger sample size.*” We were concerned that some datasets may be used by multiple investigators, and the risk of double-counting the same patients in such instances. As such, we set a rule to avoid using data from the same patients more than once by excluding studies that reported on the same dataset. In order to pick a study to exclude (if this situation arose) we decided to exclude the study that used less data (the study with a smaller sample size). As this was a relative decision (in some cases, a smaller study could involve 200 individuals, and in others 1000 individuals) we did not pre-specify a sample size threshold. As it turned out, none of our studies had overlapping cohorts.**

15. In the paragraph "We rated confidence in the reference standard as either..." describe in more detail (if possible, give examples) what ‘weak’, ‘moderate’ or ‘strong’ mean?

**Reply 15: We have revised this section to provide more detail and examples. It now reads as:**

***“ We categorized the reference standard and rated our confidence in it as either : (i) ‘weak’ when the study declared a known-group design, however its only criterion for identifying symptom exaggeration was below-chance performance on forced-choice symptom validity testing without any corroborating clinical observations or inconsistencies in medical records. For example, a patient with a mild ankle sprain is labeled as exaggerating exclusively because they fail a below‐chance forced‐choice test of pain threshold, with no clinical exam or review of documented pain or functional abilities; (ii) ‘moderate’ where most patients exaggerating symptoms were identified by forced symptom validity testing results, but some cases could be confirmed using other credible indicators. For example, a claimant insists they cannot remember simple details of their daily routine (e.g., the route to their kitchen), yet is casually observed navigating complex tasks with no apparent cognitive difficulty; or (iii) ‘strong’ where exaggeration was determined by either forced symptom validity testing results or other credible clinical evidence. For example, a clinical finding that would classify a patient presenting with persistent post-concussive complaints after a very mild head injury as exaggerating symptoms would include claims of remote memory loss (e.g., loss of spelling ability).”***

Once again, we appreciate the detailed attention to our study by the editors and reviewers and remain hopeful that our manuscript will be accepted for publication in *PLOS ONE*.

We thank you for your time and look forward to hearing from you.

Sincerely,

Andrea Darzi and Jason Busse; on behalf of all authors
